# Evaluation of a Serious Video Game to Facilitate Conversations About Human Papillomavirus Vaccination for Preteens: Pilot Randomized Controlled Trial

**DOI:** 10.2196/16883

**Published:** 2020-12-03

**Authors:** Joan R Cates, Bernard F Fuemmeler, Laurie L Stockton, Sandra J Diehl, Jamie L Crandell, Tamera Coyne-Beasley

**Affiliations:** 1 UNC Hussman School of Journalism and Media University of North Carolina at Chapel Hill Chapel Hill, NC United States; 2 Massey Cancer Center Virginia Commonwealth University Richmond, VA United States; 3 Department of Pediatrics Children's of Alabama University of Alabama Birmingham, AL United States

**Keywords:** video games, papillomavirus vaccines, adolescent health

## Abstract

**Background:**

In the United States, the most common sexually transmitted infection, human papillomavirus (HPV), causes genital warts and is associated with an estimated 33,700 newly diagnosed cancer cases annually. HPV vaccination, especially for preteens aged 11 to 12 years, is effective in preventing the acquisition of HPV and HPV-associated cancers. However, as of 2018, completion of the 2- or 3-dose HPV vaccination series increased only from 48.6% to 51.1% in teens aged 13 to 17 years, and this increase was observed only in boys. By comparison, 88.7% of teens had more than one dose of the recommended vaccine against tetanus, diphtheria, and acellular pertussis (Tdap), and 85.1% of teens had more than one dose of meningococcal vaccine. Immunizations for Tdap, meningococcal disease, and HPV can occur at the same clinical visit but often do not.

**Objective:**

Vaccination against HPV is recommended for routine use in those aged 11 to 12 years in the United States, yet it is underutilized. We aimed to develop an educational video game to engage preteens in the decision to vaccinate.

**Methods:**

*Land of Secret Gardens* is a metaphor for protecting seedlings (body) with a potion (vaccine). We screened 131 dyads of parents and preteens from 18 primary practices in North Carolina who had not initiated HPV vaccination. We measured vaccination intentions, story immersion, and game play and documented HPV vaccination rates. A total of 55 dyads were enrolled, and we randomly assigned 28 (21 completed) to play the game and 27 (26 completed) to the comparison group.

**Results:**

In total, 18 preteens reported playing the game. The vaccination self-efficacy score was higher in the comparison group than the intervention group (1.65 vs 1.45; *P*=.05). The overall mean decisional balance score trended toward greater support of vaccination, although differences between the groups were not significant.. Vaccine initiation and completion rates were higher in the intervention group (22% vs 15%; *P*=.31) than in the comparison group (9% vs 2%; *P*=.10), although the difference was not significant.

**Conclusions:**

Video games help preteens in the decision to pursue HPV vaccination. A serious video game on HPV vaccination is acceptable to parents and preteens and can be played as intended. Gamification is effective in increasing preteen interest in HPV vaccination, as game features support decision making for HPV vaccination.

**Trial Registration:**

ClinicalTrials.gov NCT04627298; https://www.clinicaltrials.gov/ct2/show/NCT04627298

## Introduction

### Background

More than a decade has passed since a vaccine to prevent human papillomavirus (HPV) infection was recommended for routine use in children aged 11 to 12 years in the United States [1]. The most common sexually transmitted infection in the United States, HPV, causes genital warts and is associated with an estimated 33,700 newly diagnosed cancer cases [2]. However, as of 2018, completion of the 2- or 3-dose HPV vaccination series increased only from 48.6% to 51.1% in teens aged 13 to 17 years, and this increase was observed only in boys [1]. By comparison, 88.7% of teens had more than 1 dose of the recommended vaccine against tetanus, diphtheria, and acellular pertussis (Tdap), and 85.1% of teens had more than 1 dose of meningococcal vaccine [2]. Immunizations for Tdap, meningococcal disease, and HPV can occur at the same clinical visit but often do not [3]. Testing and evaluating practice-based implementation strategies are needed to improve the uptake of effective interventions to increase HPV vaccination initiation and completion.

Full HPV vaccination coverage has been challenging due, in part, to providers not making strong recommendations [1]. There also remain parental concerns about the vaccine. For instance, some parents perceived the risk of HPV infection to be negligible, expressed concern about side effects, and believed the vaccine might encourage promiscuous behavior or that it may be too costly [4,5]. Health care professionals have reported that these parental attitudes and concerns are barriers to vaccination [4]. Thus, understanding and addressing these barriers would be critical to target within the context of interventions designed to increase uptake.

Although many interventions promoting HPV vaccination have focused on either the parent or provider separately with moderate success [6,7], it is increasingly being recognized that a multilevel approach may further broaden dissemination efforts [8,9]. Communication strategies have focused on giving accurate information about HPV vaccination and on training providers to give clear messages about the safety and efficacy of HPV vaccines [10].

In response to this challenge of suboptimal HPV vaccination, our interdisciplinary team (communication, public health, medicine, clinical psychology, biostatistics, health economics, and nursing) has been leading efforts to design and implement multilevel communication strategies that target parents, health care providers, and preteens [11-13]. For instance, our approach has been to develop methods to address parent resistance and misunderstandings about why the vaccine is needed early in development before their children are sexually active [14,15]. In addition, we have worked to develop strategies to address provider’s perceived barriers about discussing the vaccine with their patients (eg, helping them to develop *talking points* that can be used within a short patient visit) [16]. Finally, recognizing that serious video games may have the potential to educate preteens about sexually transmitted diseases or the utility of vaccination [17,18] and can be effective in promoting health behaviors in children and adolescents [19-23], we developed a serious video game to promote HPV vaccination among preteens [24]. Specifically, with input from preteens aged 11 to 12 years and their parents, we developed *Land of Secret Gardens*, a serious video game designed to teach about vaccines through an immersive story and to motivate a decision to seek HPV vaccination [24].

Putting all of these pieces together, we initiated the Protect Them study, which was undertaken in 36 primary care practices with 97 providers (MD, DO, NP, and RN) in North Carolina. This was a multiple baseline study and included 3 waves of activity and adjustment in 2015, 2016, and 2017. The intervention was designed to promote communication among providers, parents, and preteens to increase HPV vaccination for preteens aged 11 to 12 years when the vaccine is most effective [25]. Communication tools for the providers included brochures, posters, web-based information for parents, and interactive web-based training for providers. In addition, as part of the intervention, we provided select patients access to the Land of the Secret Garden, an age-appropriate, entertaining web-based video game designed to educate preteens about HPV infection and HPV vaccination and to promote conversations with parents and providers and the decision to vaccinate.

The video game incorporated gamification elements (eg, points, badges for completion of tasks, and a leaderboard) [26] to increase interest among players while also aiming to increase preteen knowledge and vaccine self-efficacy (ie, confidence in getting the vaccine despite barriers). Self-determination theory [27] was also used to inform the game design, as this has previously been used to evaluate the motivational pull of video games [23]. In addition, the game included an immersive story to enhance motivation to play the game [23,28] and engender deeper information processing [21]. Our hypothesis was that raising awareness about HPV vaccination eases conversations about the vaccination. We created a story about a secret garden as a metaphor for a preteen’s body and keeping it healthy. The goal was to plant a lush secret garden and protect the seedlings by treating them with a potion when they sprout to keep them healthy as they mature. Points to buy seeds and create the potion were earned by playing minigames. The minigames included several versions of finding secret objects in a garden shed and another that involved shooting down spikey balls (ie, the HPV) before they land on budding plants. Throughout the play, players were exposed to messaging about HPV and the benefits of the vaccine [24].

### Objectives

Herein*,* we report on the evaluation of the *Land of Secret Gardens* game. The aims of this pilot study are to evaluate (1) preliminary data to determine whether children who received the *Land of Secret Gardens* game had better self-reported outcomes related to HPV knowledge or vaccination self-efficacy compared with those in a control group who did not receive the game and (2) outcomes related to the game play experience (in-game autonomy and competence, presence in the game, intuitive controls, interest or enjoyment, and characteristics of playing the game) among those who received the game. We also conducted focus groups among those who received the game to further assess the acceptability of the game and whether the preteens understood the meaning of the game. Finally, we compared HPV vaccination initiation and completion among those who received the game compared with the control group who did not receive the game.

## Methods

### Participants and Procedures

Participants of this video game evaluation study were part of the lager Protect Them study mentioned earlier in the Introduction section. Parent and preteen dyads were recruited by providers at 36 different clinical sites that included family medicine practices, pediatric practices, and health departments. To enroll in the study, clinical sites in the Protect Them study agreed to identify up to 10 parents (wave 1) of preteens aged 11 to 12 years and up to 20 parents (waves 2 and 3) of preteens. Preteens aged 11 to 12 years and who had not received any doses of HPV vaccine were eligible to participate in the study. Providers contacted potential dyads via letters or telephone after they identified eligible preteens from their electronic medical records. Interested dyads provided their contact information (ie, phone number and email address) to the research team and signed a Health Insurance Portability and Accountability Act (HIPAA) release of information form to allow the research team to determine their HPV vaccination status from their health care provider. A practice champion provided a copy of the HIPAA form and the parent contact information to project staff. The most common obstacle was a high proportion of clients with parents needing informed consent provided in Spanish, an accommodation not available for this study.

After the names of eligible parents and preteens were passed to the research team, research staff invited parents to participate in a telephone conversation about informed consent with their preteen via email and telephone contacts. The study protocol required up to 3 attempts to reach parents by both email and telephone contact. Staff used an institutional review board (IRB)–approved script to conduct an informed consent procedure with the parent and then with the preteen if the parent provided consent. The script included a description of the intervention and the process of random assignment to a study group, explained the risks and benefits of participation, and reviewed the study activities and incentives for participation. Both parents and preteens were assured that their information was confidential and that participation was voluntary. In addition to preteens who had not received any HPV vaccine, eligible dyads confirmed that they had access to the internet and a mobile device or personal computer to complete the surveys and play the video game. They also provided a mailing address to receive gift certificates. Once they were enrolled, dyads were randomly assigned in a 1:1 ratio to either the intervention group or control group using a simple randomization schedule generated by the study team’s statistician. Those who were assigned to the intervention arm received the game, whereas those assigned to the control group arm did not receive the game. The study procedures were approved by the university’s nonbiomedical IRB.

**Figure 1:**
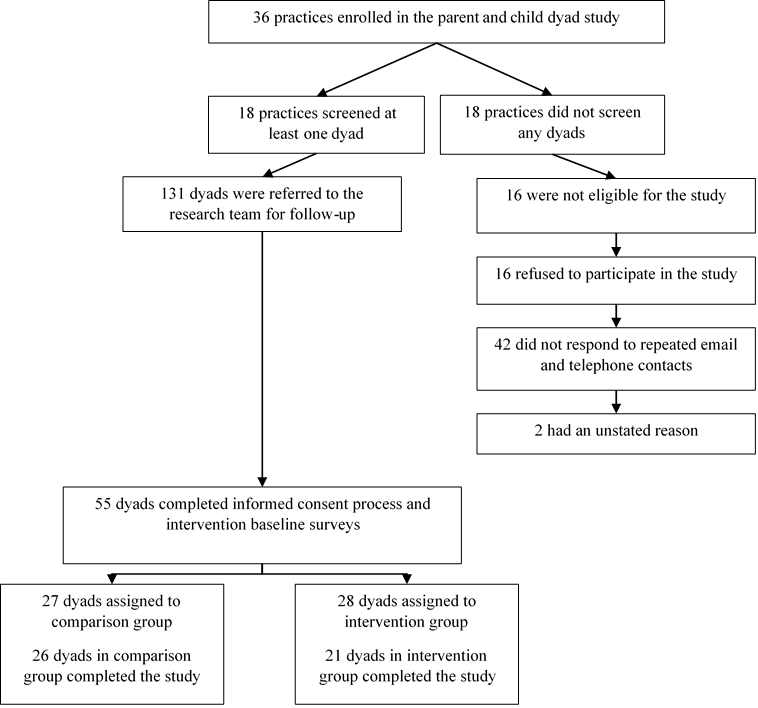
Sample results from recruitment of parent or preteen dyads through practices (n=36).

A total of 36 practices across 3 regions in North Carolina enrolled in the Protect Them study (Figure 1)—12 practices were recruited from the eastern region during wave 1 (February 2015 to June 2015), 18 practices were recruited from the central region during wave 2 (February 2016 to July 2016), and 6 practices were recruited from the western region during wave 3 (February 2017 to October 2017).

All practices were asked to screen dyads as part of the intervention. Half of the practices (18/36, 50%) screened at least one dyad, and a total of 131 dyads were referred to the research team. Of these 131 dyads, 16 (12.2%) did not meet the eligibility criteria for the study. Among the 115 that were eligible, 16 (13.9%) refused to participate in the study, and 42 (36.5%) dyads did not respond to repeated email and telephone contact. Almost half of the eligible dyads (55/115, 47.8%) completed the informed consent process and baseline surveys and were enrolled in the study, and 47 dyads completed the postsurvey.

The final study sample available for this evaluation of the game consisted of 28 preteens in the intervention group and 27 in the comparison group (21 and 26, respectively, completed the study). Preteens in the intervention group were 57% (16/28) female and 71% (20/28) white, and in the comparison group, 52% (14/27) were female and 82% (22/27) were white. The comparison group included 5 Blacks or African Americans, and the intervention group included 4 Blacks or African Americans. Two participants in each group were identified as Hispanic. The intervention group contained 9 participants aged 11 years and 19 participants aged 12 years, and the comparison group contained 14 participants aged 11 years, 11 participants aged 12 years, and 2 participants aged 13 years. There were no statistically significant differences between the groups with respect to gender, age, race, and Hispanic ethnicity.

#### Survey Procedures

We asked all parents and preteens to complete the baseline and postintervention surveys. The surveys were designed in Qualtrics [29], and survey links were sent to the parent’s email address. Dyads received the baseline surveys before potential exposure to the video game and parent portal. Up to 5 email reminders and 3 telephone calls were made to encourage survey completion. Follow-up reminders were offered to encourage game play and completion of the task. Each participant received a US $25 gift certificate to Walmart if they completed a survey.

Study groups were asked identical questions about their knowledge and attitudes about HPV and their intentions to vaccinate against HPV at baseline. Postintervention surveys were sent 4 months after participants completed their baseline surveys. All participants were asked if the preteen received any dose of the HPV vaccine. Participants in the intervention group were asked additional questions about their experience with the intervention and the Protect Them resources.

#### Game Play Procedures

Preteens in the intervention group were asked to play the *Land of Secret Gardens* and complete 3 tasks in the video game. The tasks occurred in a sequence that required the player to return to the game multiple times. A badge appeared on the leaderboard on completion of each task, which allowed preteens to track their progress. Preteens were exposed to messages about HPV and the HPV vaccination throughout the game, and continued use of the game would result in greater message exposure. Project staff monitored the game play progress for each participant with Navicat [30] and sent reminders to parents to encourage game play. Instructions for parents to guide game play were posted on the parent portal, along with a video that described the background and premise of the game. A help form was also available on the parent portal to request technical support for the game.

### Measures

We assigned preteens in the intervention group to play the video game and asked their parents to review web-based materials and then collected baseline and postintervention data from both study groups via Qualtrics surveys. We measured knowledge, vaccination self-efficacy, and decisional balance in both groups. From the intervention group only, we collected data on Physical/Emotional/Narrative Presence Scale (PENS) [31] to gauge preteens’ immersion in the game. Finally, from the practice champion, we collected the HPV vaccination status of each preteen participant approximately 9 months postintervention.

The description of each measure is as follows:

- *Knowledge scale*: asked in both the intervention and comparison groups. The 5 items asked whether HPV vaccination (1) can prevent genital warts, (2) can prevent cervical cancer, (3) can prevent anal cancer, (4) can prevent throat cancer, and (5) is recommended for 11- and 12-year-old boys and girls.
- *Vaccination Self-efficacy and decisional balance scales* [32]: used in the intervention group and the comparison group to compare self-efficacy and intentions to vaccinate and decisional balance. We used 8 items for vaccination self-efficacy, 4 items for positive decision to vaccinate, and 5 items for negative decision to vaccinate. All questions were rated on a 3-point Likert scale (1=not at all, 2=somewhat, and 3=a lot).
- *PENS* [31]: to learn more about the intervention experience, we collected measures specific to preteens in the intervention group. We used measurements from PENS to gauge the extent of the preteen’s immersion in the story.
- *Game play*: we asked participants who played the game how they played the game, for example, earning at least one shield, playing 3 or more times, playing more than 10 min per session, playing with a parent or sibling, playing the shield game and the hidden objects game, creating a potion, and correctly identifying the game metaphor. In addition, we recruited 3 postintervention focus groups with preteens (2 or 3 in each group) who played the game and asked about their experience. The focus groups were conducted and recorded via telephone calls. Parents gave consent before the preteens joined the conversation. The study moderator asked whether the preteens enjoyed the game and which parts they enjoyed or did not enjoy.
- *HPV immunization records*: obtained for all preteen participants from practice champions approximately 9 months following each intervention. This period allowed enough interval for the preteens to complete their HPV vaccination series.

### Statistical Analysis

We compared postintervention knowledge, vaccination self-efficacy, and decisional balance in the intervention and comparison groups using two-sample *t* tests, with significance level of α=.05. Immunization records were obtained for all preteen participants approximately 9 months postintervention for each cohort total. During the time of the study, the HPV vaccine was offered at both a 2-dose schedule with up to 6 to 12 months after the first shot and a 3-dose schedule with up to 6 months after the first shot. Practice champions were asked whether a preteen was on a 2- or 3-dose HPV vaccination schedule and to confirm whether they initiated and/or completed the vaccine series. The intervention and comparison groups were compared regarding the initiation and completion of the vaccine series using the Mantel-Haenszel chi-square test stratified by intervention wave.

## Results

### Knowledge and Vaccination Self-Efficacy and Decisional Balance

Postintervention, the mean knowledge score (5 items, range 1-3) was higher in the intervention group than in the comparison group (2.56, SD 0.34 vs 2.28, SD 0.41 respectively; *P=*.03)*.* The results of the self-efficacy and decisional balance results are given in Table 1 and summarized as follows.

**Table 1:** Mean comparisons between items from the self-efficacy and decisional balance scales at post-intervention surveys.

Items from self-efficacy and decisional balance scales			Intervention (n=21), mean (SD)	Comparison (n=26), mean (SD)	*P* value
**Vaccination self-efficacy. How confident am I about getting the vaccine…**					
	When I think about the possible side effects of the vaccine?		1.77 (0.69)	1.85 (0.61)	.70
	When I think that the shot will be painful?		1.91 (0.75)	1.92 (0.74)	.95
	When my parents are getting me vaccinated?		1.29 (0.56)	1.46 (0.65)	.33
	When I think I will faint or get dizzy when getting the shot?		1.55 (0.74)	1.46 (0.71)	.69
	When it is too expensive?		1.18 (0.5)	1.54 (0.65)	.04
	When it is too inconvenient?		1.23 (0.53)	1.73 (0.60)	.01
	When the doctor does not strongly recommend it?		1.41 (0.67)	1.62 (0.64)	.28
	When my friends will know I got the shot?		1.23 (0.53)	1.62 (0.75)	.04
All vaccination self-efficacy items			1.45 (0.35)	1.65 (0.35)	.05
**Importance. How important is this item in deciding to get HPV^a^ vaccination?**					
	**Pros**				
		Protecting myself from HPV would make me feel good.	2.55 (0.51)	2.39 (0.50)	.31
		I would be protected from certain cancers and genital warts.	2.57 (0.51)	2.55 (0.51)	.87
		I would be protecting myself from getting a sexually transmitted infection	2.36 (0.58)	2.31 (0.74)	.77
		I would be less likely to spread HPV	2.19 (0.81)	1.92 (0.8)	.26
	All pros items		2.44 (0.39)	2.31 (0.42)	.29
	**Cons**				
		Receiving the series would take too much time	1.77 (0.69)	1.73 (0.72)	.84
		It would be too embarrassing to talk to my parents	1.41 (0.59)	1.31 (0.55)	.54
		It would be too embarrassing to talk to my doctor about getting vaccinated	1.55 (0.6)	1.5 (0.65)	.80
		My parents would not approve of me receiving the vaccine	1.1 (0.31)	1.62 (0.8)	.01
		My parents would think I was having sex if I got vaccinated	1.0 (0.01)	1.19 (0.57)	.13
	All cons items		1.38 (0.31)	1.47 (0.43)	.39
Decisional balance (pros-cons)			1.07 (0.59)	0.82 (0.63)	.18

^a^HPV: human papillomavirus.

The mean vaccination self-efficacy score was higher in the comparison group than in the intervention group (1.65 vs 1.45, respectively; *P*=.05). Only 3 of the 8 individual items in the scale were significantly different and were in the direction of *confident—not at all*: *When it is too expensive*? *When it is too inconvenient?* *When my friends will know I got the shot?* The overall mean decisional balance score trended toward greater support of vaccination, although differences between the groups were not significant. As seen in Table 1, *Pros* vaccination scores were higher in the intervention group than in the comparison group (2.44 vs 2.31, respectively), and *cons* vaccination scores were lower in the intervention group than in the comparison group (1.38 vs 1.48, respectively), but again these were not statistically significant differences between the groups.

### PENS

The 18 participants who reported on measures of physical/emotional/narrative presence in the game [31] gave mixed reviews on the game. More than half of the participants gave positive scores on game autonomy and competence, ease, and freedom of playing the game (Table 2). At the same time, more than half of the participants called the game boring and said they were not impacted emotionally and that the game did not hold their attention. Thus, the results of this scale revealed both positive and negative evaluations of the game.

**Table 2:** Measurements from the Physical/Emotional/Narrative Presence Scale (N=18).

Characteristics		Participants who agree or strongly agree, n (%)
**In-game autonomy**		
	The game provides me with interesting options and choices	7 (39)
	The game lets you do interesting things	13 (72)
	I experienced a lot of freedom in the game	9 (50)
**In-game competence**		
	I feel competent at the game	7 (39)
	I feel very capable and effective when playing	8 (44)
	My ability to play the game is well-matched with the game's challenges	8 (44)
**PENS^a^**		
	When playing the game, I feel transported to another time and place	4 (22)
	Exploring the game world feels like taking an actual trip to a new place	5 (28)
	When moving through the game world, I feel as if I am actually there	3 (17)
	I am not impacted emotionally by events in the game (−)	9 (50)
	The game was emotionally engaging	4 (22)
	I experience feelings as deeply in the game as I have in real life	4 (22)
	When playing the game, I feel as if I was part of the story	4 (22)
	When I accomplished something in the game I experienced genuine pride	6 (33)
	I had reactions to events and characters in the game as if they were real	1 (6)
**PENS: intuitive controls**		
	Learning the game controls was easy	10 (56)
	The game controls are intuitive	5 (28)
	When I wanted to do something in the game, it was easy to remember the corresponding control	10 (56)
**Postexperimental Intrinsic Motivation Inventory: interest or enjoyment** [23]		
	I enjoyed doing this game very much	7 (39)
	This game was fun to do	6 (33)
	I thought this was a boring game	9 (50)
	This game did not hold my attention at all	10 (56)
	I would describe this game as very interesting	4 (22)
	I thought this game was quite enjoyable	5 (28)
	While I was doing this game, I was thinking about how much I enjoyed it	4 (22)
	Given the chance I would play this game in my free time	4 (22)
	I would like to spend more time playing this game	4 (22)

^a^PENS: Physical/Emotional/Narrative Presence Scale.

### Game Play in the Intervention Group

Of the 21 participants assigned to the intervention group, 86% (18) reported playing the game (Table 3).

Preteens who did not play the game reported that they had technical difficulties (n=2), and a parent determined *it was not for me* (n=1). Among the preteens who played the game, 78% (14/18) reported that they played it 3 or more times. The majority of players (14/18, 78%) spent more than 10 min on the game at each session. Although the game was designed primarily for mobile devices (tablets and cellular phones), the majority of the preteens (11/18, 61%) played the game on a personal computer. Half of the participants (9/18, 50%) saved the game to their device. Fifteen of the preteens played the game with a parent, and 4 preteens played with a sibling.

**Table 3:** Self-reported characteristics of video game play among the intervention group (18 preteens who played the game).

Characteristics	Values, n (%)
Preteen played video game with a sibling	4 (22)
Smartphone or tablet used for video game play	6 (33)
Saved video game to device	9 (50)
Earned at least one shield on the leaderboard	12 (67)
Earned shields on garden plants	8 (44)
Played 3 or more times	14 (78)
Played game more than 10 minutes per session	14 (78)
Preteen played video game with a parent	15 (83)
Played the shield game	13 (72)
Played the hidden objects activity	15 (83)
Created a potion	16 (89)
Correctly identified game metaphor	16 (89)
Playing the game changed how I feel about getting the vaccine	4 (22)
I was interested in finding out more about the HPV^a^ vaccine after playing the game	6 (33)
I would recommend this game to a friend who wanted to learn about HPV	6 (33)
I was more confident to talk with a parent about the vaccine after playing the game	8 (44)
I was more willing to get the vaccine after playing the game	9 (50)
I know more about the vaccine	12 (67)

^a^HPV: human papillomavirus.

The participants were asked to complete 3 game activities: (1) play a shield game with blue spikey virus balls, (2) find hidden objects in 4 different rooms, and (3) create a potion (Multimedia Appendix 1). Nearly all the preteens (n=16) found hidden objects and created a potion, and 13 played the shield game. The preteens were able to track their progress on a leaderboard, and 12 of them reported that they earned at least one shield on their leaderboard. The game is completed when shields appear on plants in the garden. Less than half of the preteens (n=8) reported that they saw at least one shield in their garden. All but 2 preteens identified the idea behind the secret garden metaphor.

### Postintervention Focus Group

We conducted 3 focus groups with 7 preteens following each of the 3 waves in the intervention. The preteens generally enjoyed and understood the game, especially playing the hidden objects game and earning points to plant their gardens. They acknowledged that playing the game helped them to be more aware of HPV. Participants were curious about what would happen to them if they were vaccinated. They described the game as “just a game where you… just plant flowers in the garden and make a shield to protect the plants,” and “...it’s a game to help me understand about the HPV shot and what you do in the game.” Participants liked the “hidden figures game... were fun to try to find.” They said the game was easy to play and that it was fun.

The preteens remembered that messages appeared in the game, but they could not remember specific messages. Study participation did not impact preteens’ attitudes about HPV vaccination, and they agreed that playing the game made them more aware of HPV as an infection. In terms of designing the next level of the video game, they suggested more hidden objects with a higher level of difficulty and a bigger garden as well as pulling weeds out of the garden to take care of the plants. They would include more activities beyond the shield game and the hidden objects.

### HPV Vaccination Initiation Rate

The vaccine initiation rate was higher in the intervention group than in the control group, but this difference was not statistically significant (22% vs 15%, respectively; *P*=.31). Vaccination completion rates were also higher in the intervention group than in the control group (9% vs 2%, respectively; *P*=.10). Although this is not significant, it is noteworthy that only 1 of the 27 comparison group members completed the HPV vaccine series, whereas 5 of 28 intervention group members completed the HPV vaccine series. It should also be noted that most of those who initiated were still on schedule to complete, but the date for the next dose had not yet arrived when data were collected.

## Discussion

### Principal Findings

Of the 36 practices in the study, 18 were able to identify and screen parents and preteens for a total of 55 dyads. The study sample consisted of 21 preteens in the intervention group and 26 preteens in the comparison group who completed the follow-up survey. The objective of our study is to evaluate the acceptability and feasibility of using a serious video game about HPV vaccination with preteens and parents to promote conversations about and decisions to seek HPV vaccination. The scores for preteen HPV vaccination self-efficacy in our study indicated greater support postintervention for the comparison group compared with the intervention group. Only 3 of the 8 individual items were significant and in the direction of lower self-esteem. One plausible explanation for higher scores in the comparison group is that they were less aware of barriers to HPV vaccination, including expense, inconvenience, and their friends knowing that they would get the vaccine. In addition, game play was mostly positive, with more than half of the participants playing the game as intended and wanting to learn more about HPV vaccination. Less positive comments were made about not changing how they felt about the vaccine or not recommending the game to family or a friend. A greater proportion of preteens in the intervention group initiated the vaccine and had higher completion rates than their counterparts in the control group, but these differences did not reach statistical significance.

From our research and that of others, modifiable determinants to increase HPV vaccination for preteens aged 11 to 12 years include (1) knowledge, attitudes, and beliefs of parents, providers, and preteens; (2) parents’ concerns that preteen children are too young to receive vaccination and are not sexually active yet, that the vaccine is not safe, and that they do not have a vaccine recommendation from their doctor; (3) preteens’ dislike of shots and being minimally involved in the vaccination decision; and (4) providers’ concerns about parents’ resistance to vaccination, vaccine cost, and duration [33,34]. Gamification has the potential to increase engagement with health messaging relevant to shaping motivation and behavior, such as seeking HPV vaccination [21]. Gamification includes techniques to increase knowledge and shape attitudes about HPV vaccination. These techniques often provoke positive effects, depending on how they are being implemented and used [21]. The use of a garden metaphor, for example, in visualizing the importance of HPV vaccination, facilitates the preteen’s conception of a beneficial medical procedure to prevent harmful viruses. In the case of the *Land of Secret Gardens*, the challenge is to grow a healthy garden, protected from viruses.

The strengths of our study include using a serious video game to motivate interest in HPV vaccination and to promote conversations with parents, family members, and friends. We conducted focus groups with preteens and learned their viewpoints about serious video games. We further conducted focus groups with preteens as we built the game to determine functionality. Finally, we conducted focus groups after game play to learn what worked well and what did not work so well. Our thorough process will help make the game more relevant to the preteens.

### Limitations

The small sample size was a primary weakness of this study. Recruitment and retention were barriers throughout the study. Once clinic staff provided names of potential participants to research staff, follow-up with the parents via our protocol of 3 attempts via both email and telephone contact remained to be a challenge. One obstacle was a high proportion of clients with parents needing informed consent provided in Spanish, an accommodation not available for this study. Another obstacle reflected some of the measurements we had available. For instance, the decisional balance measure was developed with older subjects (college-aged women); therefore, some of the items had to be modified for use in a young population of children aged 11 to 12 years. A further limitation was relying on vaccination and self-efficacy results from a group of older teenagers. This might have skewed the results from younger teenagers.

### Conclusions

A serious video game on HPV vaccination is acceptable to parents and preteens and can be played as intended. Gamification can be effective in shaping attitudes about the HPV vaccination. Further research is needed to enhance the game with puzzles and activities that are engaging to the preteen population.

## Appendix

Multimedia Appendix 1 Video game for preteens.

Multimedia Appendix 2 CONSORT-EHEALTH checklist (V 1.6.1).

## Abbreviations

HIPAA: Health Insurance Portability and Accountability Act
HPV: human papillomavirus
IRB: institutional review board
PENS: Physical/Emotional/Narrative Presence Scale
Tdap: tetanus, diphtheria, and acellular pertussis

## References

[1] Walker TY, Elam-Evans LD, Yankey D, Markowitz LE, Williams CL, Fredua B, Singleton JA, Stokley S (2019). National, regional, state, and selected local area vaccination coverage among adolescents aged 13-17 years - United States, 2018. MMWR Morb Mortal Wkly Rep.

[2] Walker TY, Elam-Evans LD, Yankey D, Markowitz LE, Williams CL, Mbaeyi SA, Fredua B, Stokley S (2018). National, regional, state, and selected local area vaccination coverage among adolescents aged 13-17 years - United States, 2017. MMWR Morb Mortal Wkly Rep.

[3] Vielot NA, Butler AM, Brookhart MA, Becker-Dreps S, Smith JS (2017). Patterns of use of human papillomavirus and other adolescent vaccines in the United States. J Adolesc Health.

[4] Holman DM, Benard V, Roland KB, Watson M, Liddon N, Stokley S (2014). Barriers to human papillomavirus vaccination among US adolescents: a systematic review of the literature. JAMA Pediatr.

[5] Thompson EL, Rosen BL, Vamos CA, Kadono M, Daley EM (2017). Human papillomavirus vaccination: what are the reasons for nonvaccination among US adolescents?. J Adolesc Health.

[6] Niccolai LM, Hansen CE (2015). Practice- and community-based interventions to increase human papillomavirus vaccine coverage: a systematic review. JAMA Pediatr.

[7] Smulian EA, Mitchell KR, Stokley S (2016). Interventions to increase HPV vaccination coverage: a systematic review. Hum Vaccin Immunother.

[8] Mercer SL, DeVinney BJ, Fine LJ, Green LW, Dougherty D (2007). Study designs for effectiveness and translation research :identifying trade-offs. Am J Prev Med.

[9] Dempsey AF, Pyrznawoski J, Lockhart S, Barnard J, Campagna EJ, Garrett K, Fisher A, Dickinson LM, O'Leary ST (2018). Effect of a health care professional communication training intervention on adolescent human papillomavirus vaccination: a cluster randomized clinical trial. JAMA Pediatr.

[10] Markowitz LE, Gee J, Chesson H, Stokley S (2018). Ten years of human papillomavirus vaccination in the United States. Acad Pediatr.

[11] Cates JR, Crandell JL, Diehl SJ, Coyne-Beasley T (2018). Immunization effects of a communication intervention to promote preteen HPV vaccination in primary care practices. Vaccine.

[12] Cates JR, Diehl SJ, Crandell JL, Coyne-Beasley T (2014). Intervention effects from a social marketing campaign to promote HPV vaccination in preteen boys. Vaccine.

[13] Cates JR, Shafer A, Diehl SJ, Deal AM (2011). Evaluating a county-sponsored social marketing campaign to increase mothers' initiation of HPV vaccine for their pre-teen daughters in a primarily rural area. Soc Mar Q.

[14] Cates JR, Ortiz R, Shafer A, Romocki LS, Coyne-Beasley T (2012). Designing messages to motivate parents to get their preteenage sons vaccinated against human papillomavirus. Perspect Sex Reprod Health.

[15] Shafer A, Cates JR, Diehl SJ, Hartmann M (2011). Asking mom: formative research for an HPV vaccine campaign targeting mothers of adolescent girls. J Health Commun.

[16] Cates JR, Diehl SJ, Fuemmeler BF, North SW, Chung RJ, Hill JF, Coyne-Beasley T (2020). Toward optimal communication about HPV vaccination for preteens and their parents: evaluation of an online training for pediatric and family medicine health care providers. J Public Health Manag Pract.

[17] Patchen L, Ellis L, Ma TX, Ott C, Chang KH, Araya B, Atreyapurapu S, Alyusuf A, Gaines Lanzi R (2020). Engaging African American youth in the development of a serious mobile game for sexual health education: mixed methods study. JMIR Serious Games.

[18] Eley CV, Young VL, Hayes CV, Verlander NQ, McNulty CA (2019). Young people's knowledge of antibiotics and vaccinations and increasing this knowledge through gaming: mixed-methods study using e-bug. JMIR Serious Games.

[19] Thompson D, Baranowski T, Buday R, Baranowski J, Thompson V, Jago R, Griffith MJ (2010). Serious video games for health how behavioral science guided the development of a serious video game. Simul Gaming.

[20] Lu AS, Baranowski J, Islam N, Baranowski T (2014). How to engage children in self-administered dietary assessment programmes. J Hum Nutr Diet.

[21] Baranowski T, Buday R, Thompson DI, Baranowski J (2008). Playing for real: video games and stories for health-related behavior change. Am J Prev Med.

[22] Thompson D, Baranowski T, Buday R (2010). Conceptual model for the design of a serious video game promoting self-management among youth with type 1 diabetes. J Diabetes Sci Technol.

[23] Ryan RM, Rigby CS, Przybylski A (2006). The motivational pull of video games: a self-determination theory approach. Motiv Emot.

[24] Cates JR, Fuemmeler BF, Diehl SJ, Stockton LL, Porter J, Ihekweazu C, Gurbani AS, Coyne-Beasley T (2018). Developing a serious videogame for preteens to motivate HPV vaccination decision making: land of secret gardens. Games Health J.

[25] Meites E, Kempe A, Markowitz LE (2016). Use of a 2-dose schedule for human papillomavirus vaccination - updated recommendations of the advisory committee on immunization practices. MMWR Morb Mortal Wkly Rep.

[26] (2019). Gamification. Merriam Webster Inc.

[27] Ryan D (2000). Intrinsic and extrinsic motivations: classic definitions and new directions. Contemp Educ Psychol.

[28] McGonigal J (2011). Reality is Broken : Why Games Make Us Better and How They Can Change the World.

[29] (2018). Survey Research Suite. Qualtrics Inc.

[30] (2018). Navicat Database Management.

[31] Busselle R, Bilandzic H (2009). Measuring narrative engagement. Media Psychol.

[32] Lipschitz JM, Fernandez AC, Larson HE, Blaney CL, Meier KS, Redding CA, Prochaska JO, Paiva AL (2013). Validation of decisional balance and self-efficacy measures for HPV vaccination in college women. Am J Health Promot.

[33] (2013). National and State Vaccination Coverage Among Adolescents Aged 13–17 Years — United States, 2011. Centers for Disease Control and Prevention.

[34] Brewer NT, Gottlieb SL, Reiter PL, McRee AL, Liddon N, Markowitz L, Smith JS (2011). Longitudinal predictors of human papillomavirus vaccine initiation among adolescent girls in a high-risk geographic area. Sex Transm Dis.

