# Supplementary material for: Evaluation of a Serious Video Game to Facilitate Conversations About Human Papillomavirus Vaccination for Preteens: Pilot Randomized Controlled Trial
Source: JMIR Serious Games. 2020 Dec 3;8(4):e16883. doi: 10.2196/16883 (PMC7746502; doi:10.2196/16883)

## Slide 1
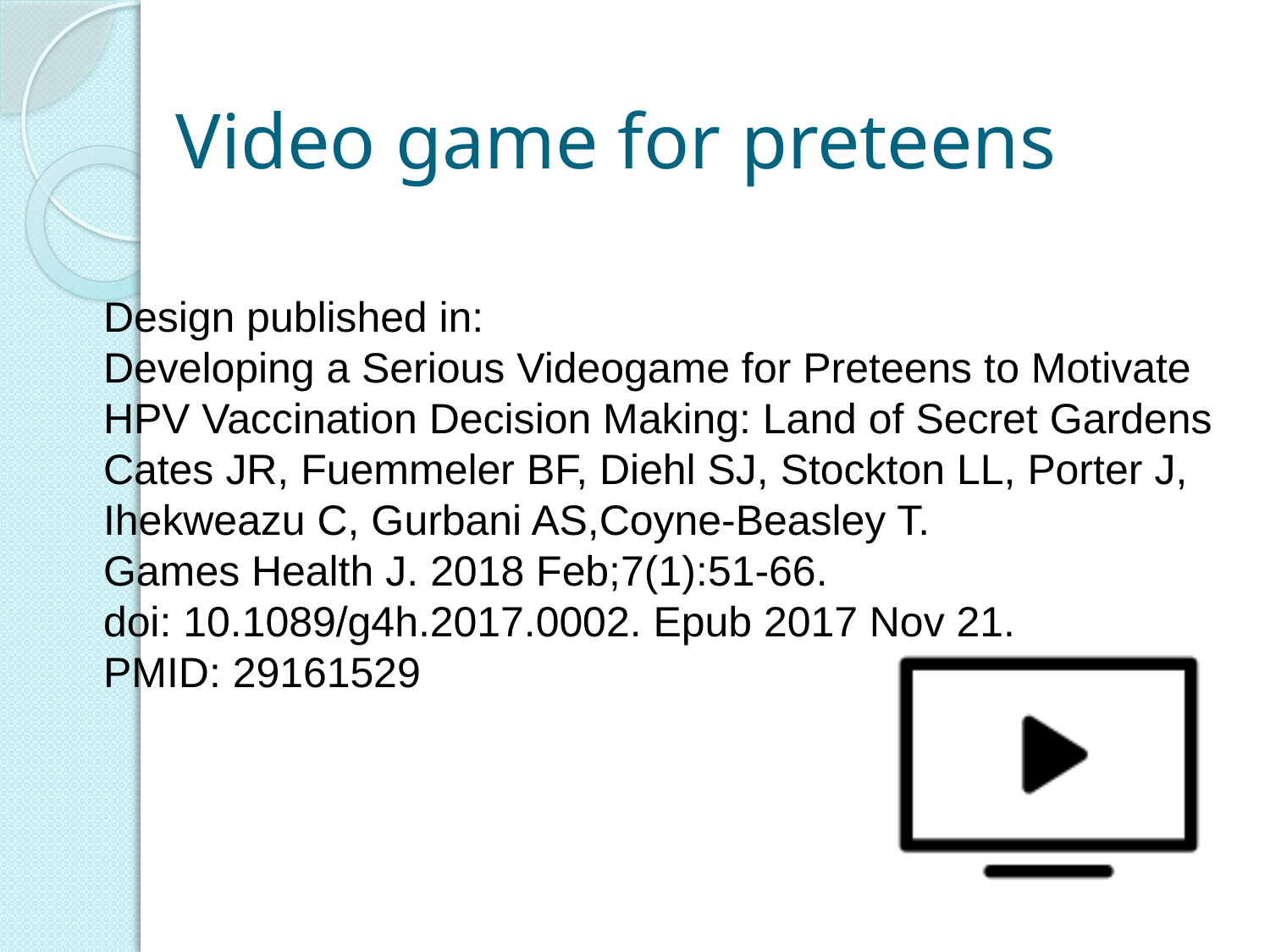

# Video game for preteens
Design published in:
Developing a Serious Videogame for Preteens to Motivate
HPV Vaccination Decision Making: Land of Secret Gardens
Cates JR, Fuemmeler BF, Diehl SJ, Stockton LL, Porter J,
Ihekweazu C, Gurbani AS,Coyne-Beasley T.
Games Health J. 2018 Feb;7(1):51-66.
doi: 10.1089/g4h.2017.0002. Epub 2017 Nov 21.
PMID: 29161529

## Slide 2
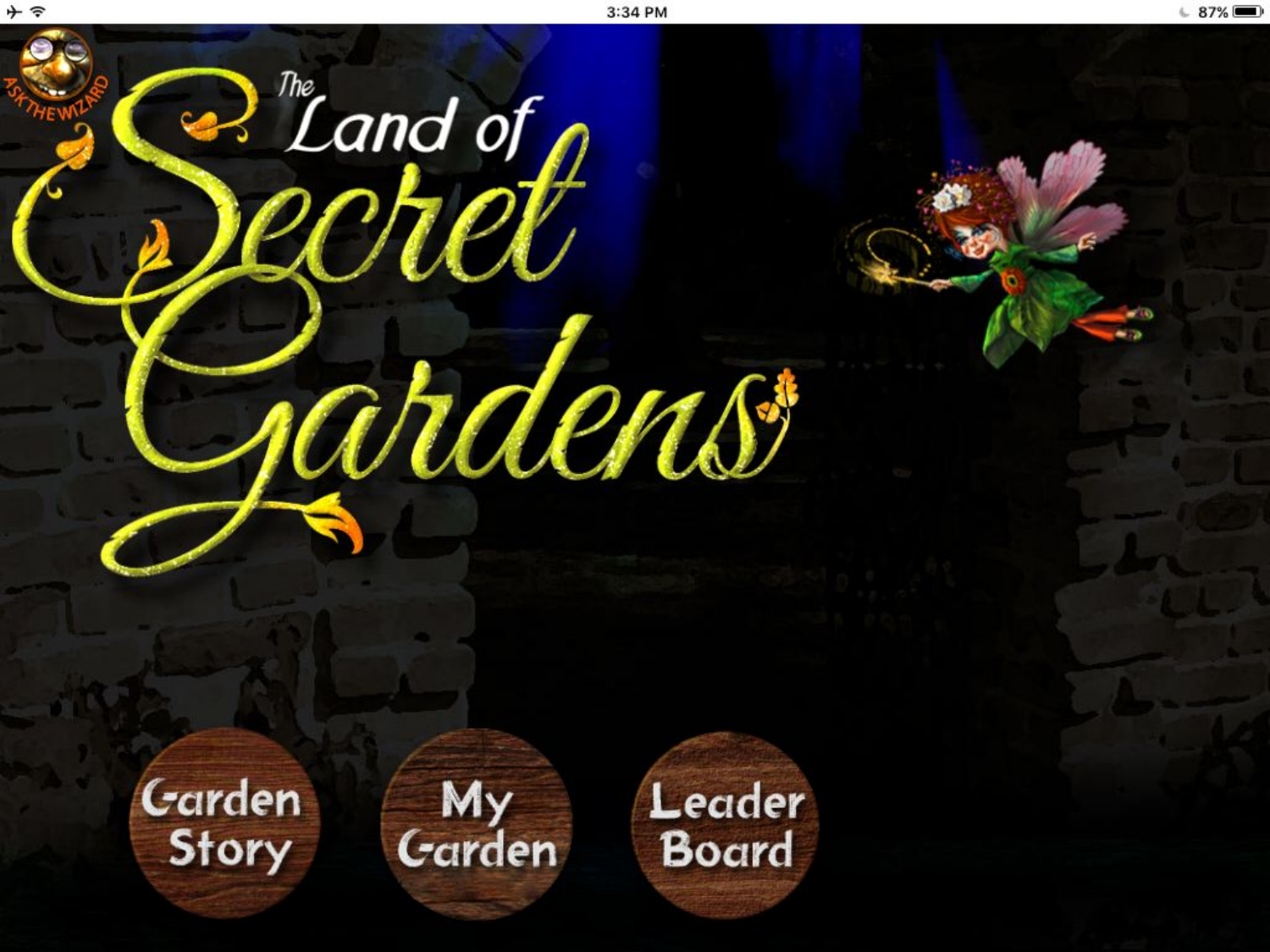

## Slide 3
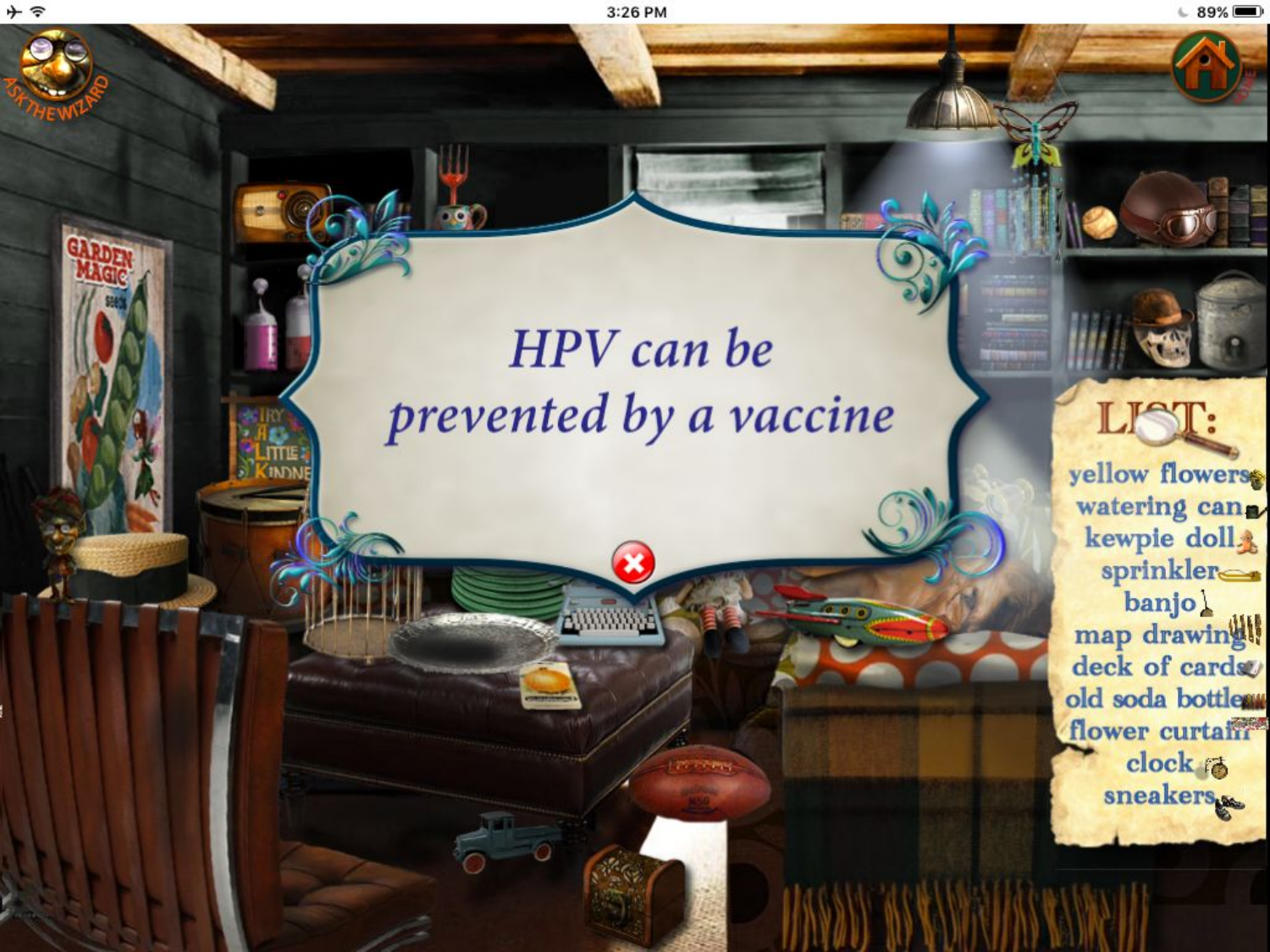

## Slide 4
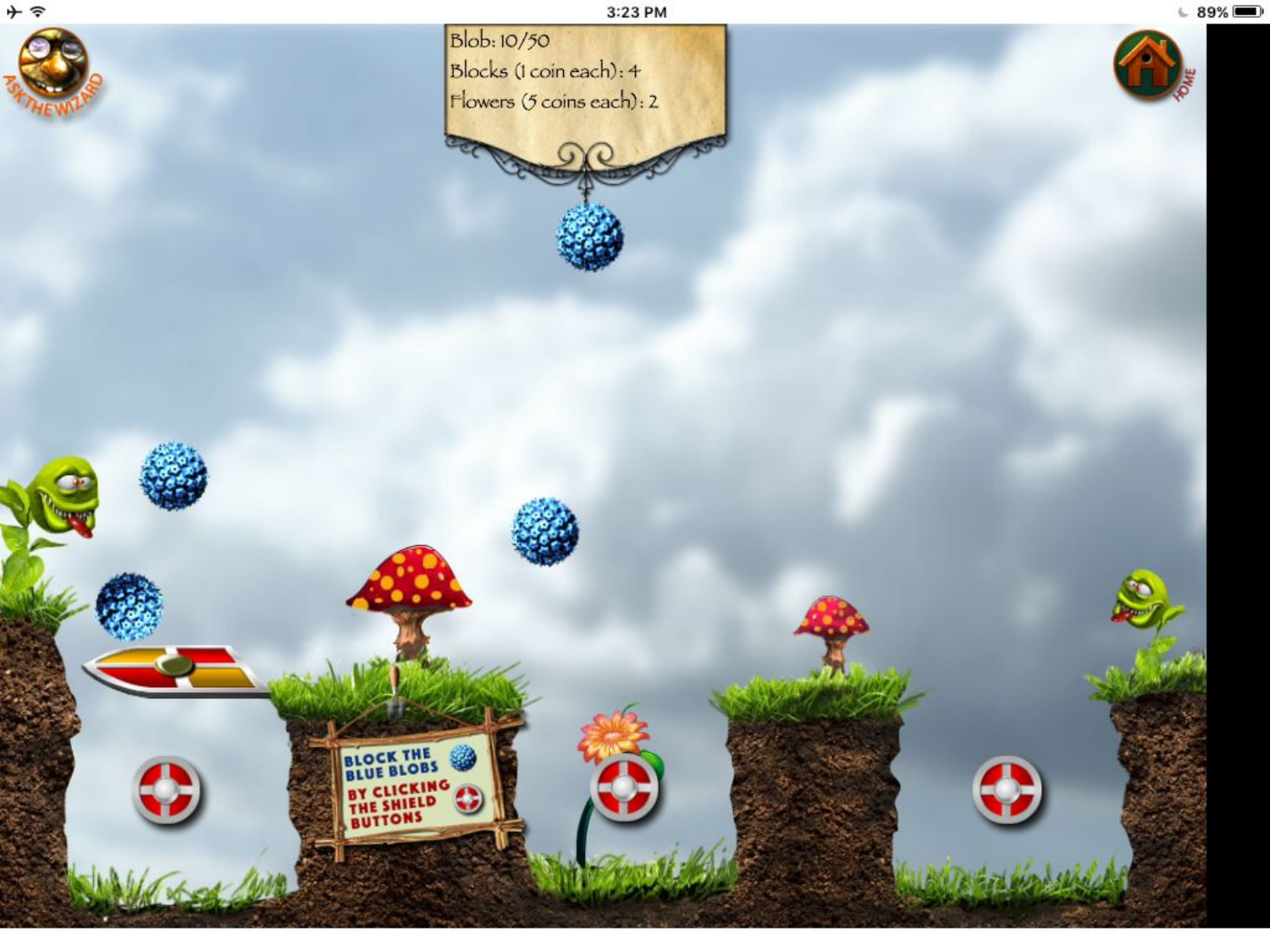

## Slide 5
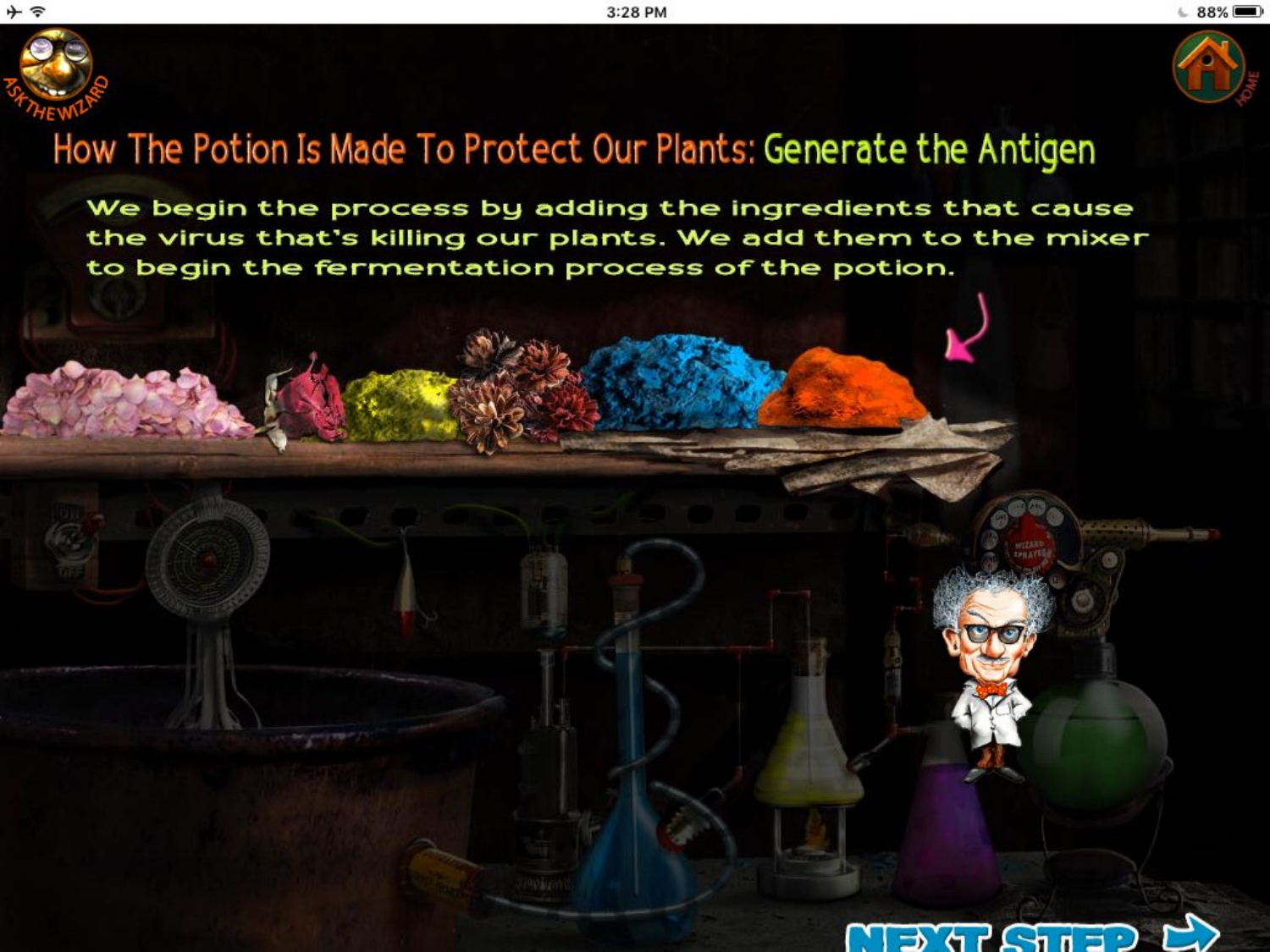

## Slide 6
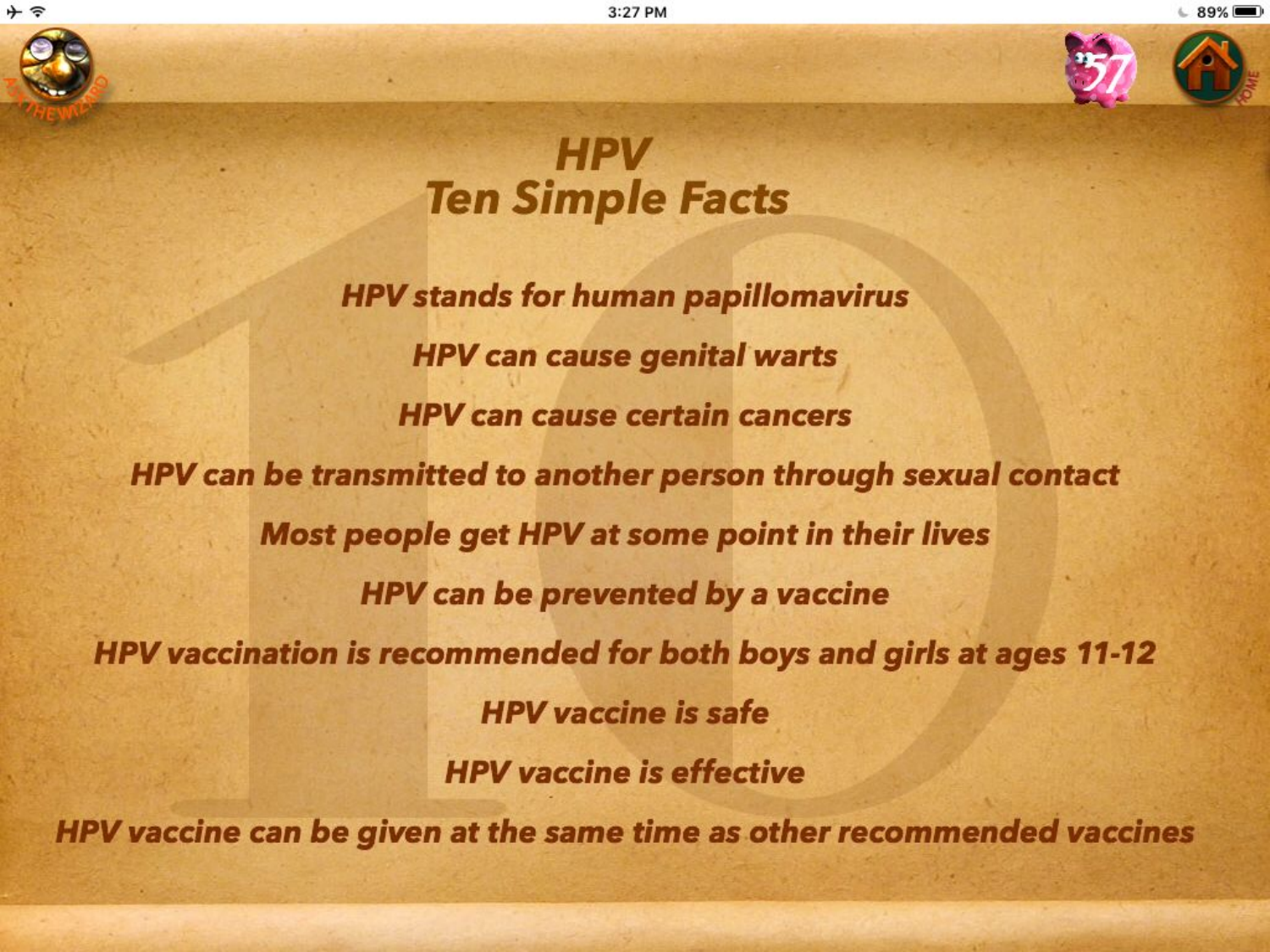

## Slide 7
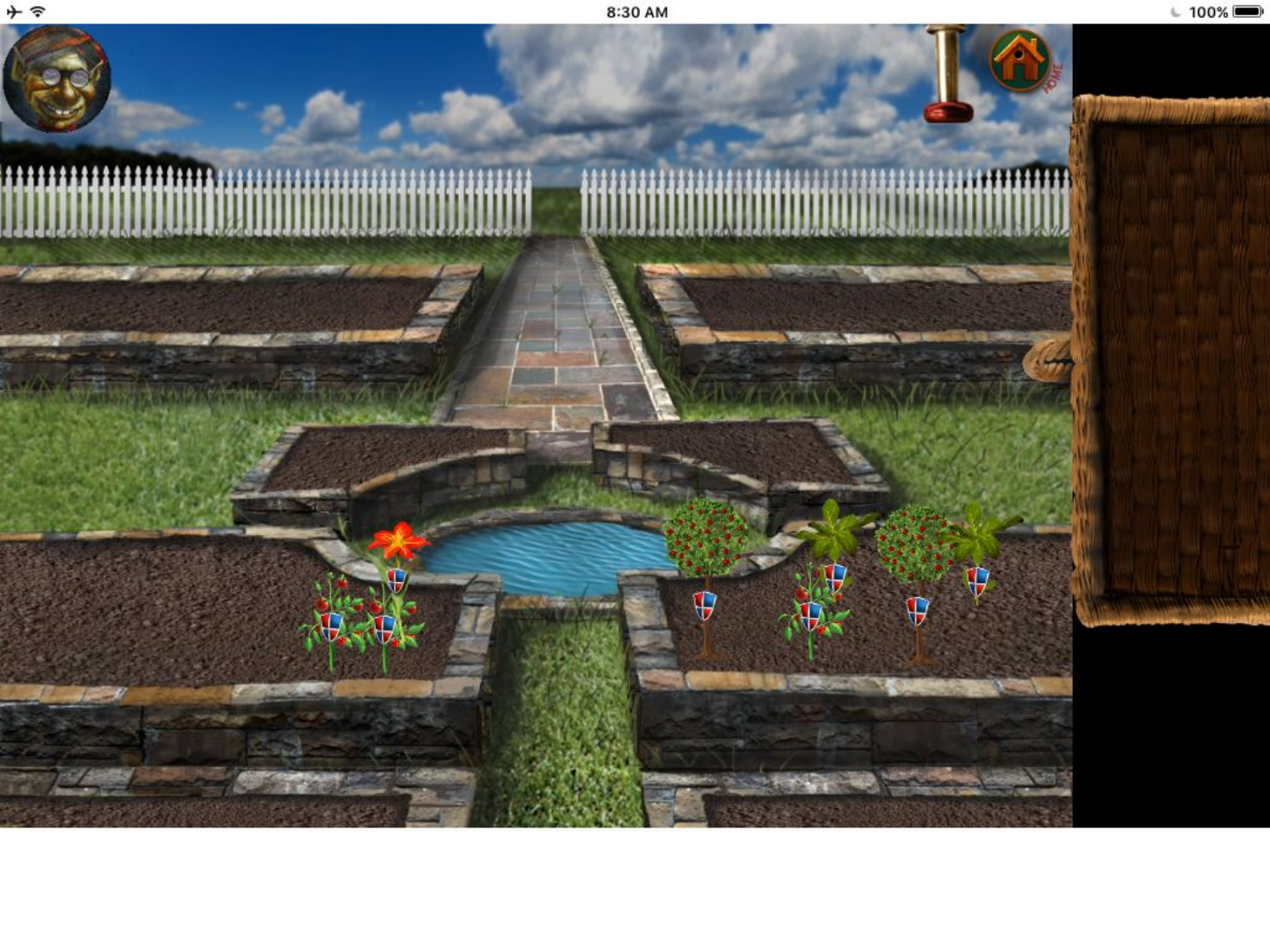

Supplement: Multimedia Appendix 1 [file games_v8i4e16883_app1.pptx]
