# Supplementary material for: Evaluation of a Serious Video Game to Facilitate Conversations About Human Papillomavirus Vaccination for Preteens: Pilot Randomized Controlled Trial
Source: JMIR Serious Games. 2020 Dec 3;8(4):e16883. doi: 10.2196/16883 (PMC7746502; doi:10.2196/16883)
Supplement: Multimedia Appendix 2 [file games_v8i4e16883_fig.pdf]

CONSORT-EHEALTH (V 1.6.1) - Submission/Publication Form
The CONSORT-EHEALTH checklist is intended for authors of randomized trials evaluating web-based and Internet-based applications/interventions, including mobile interventions, electronic games (incl multiplayer games), social media, certain telehealth applications, and other interactive and/or networked electronic applications. Some of the items (e.g. all subitems under item 5 - description of the intervention) may also be applicable for other study designs.

The goal of the CONSORT EHEALTH checklist and guideline is to be
a) a guide for reporting for authors of RCTs,
b) to form a basis for appraisal of an ehealth trial (in terms of validity)

CONSORT-EHEALTH items/subitems are MANDATORY reporting items for studies published in the Journal of Medical Internet Research and other journals / scientific societies endorsing the checklist.

Items numbered 1., 2., 3., 4a., 4b etc are original CONSORT or CONSORT-NPT (non-pharmacologic treatment) items.
Items with Roman numerals (i., ii, iii, iv etc.) are CONSORT-EHEALTH extensions/clarifications.

As the CONSORT-EHEALTH checklist is still considered in a formative stage, we would ask that you also RATE ON A SCALE OF 1-5 how important/useful you feel each item is FOR THE PURPOSE OF THE CHECKLIST and reporting guideline (optional).

Mandatory reporting items are marked with a red *.
In the textboxes, either copy & paste the relevant sections from your manuscript into this form - please include any quotes from your manuscript in QUOTATION MARKS,
or answer directly by providing additional information not in the manuscript, or elaborating on why the item was not relevant for this study.

YOUR ANSWERS WILL BE PUBLISHED AS A SUPPLEMENTARY FILE TO YOUR PUBLICATION IN JMIR AND ARE CONSIDERED PART OF YOUR PUBLICATION (IF ACCEPTED).
Please fill in these questions diligently. Information will not be copyedited, so please use proper spelling and grammar, use correct capitalization, and avoid abbreviations.

DO NOT FORGET TO SAVE AS PDF _AND_ CLICK THE SUBMIT BUTTON SO YOUR ANSWERS ARE IN OUR DATABASE !!!

Citation Suggestion (if you append the pdf as Appendix we suggest to cite this paper in the caption):
Eysenbach G, CONSORT-EHEALTH Group
CONSORT-EHEALTH: Improving and Standardizing Evaluation Reports of Web-based and Mobile Health Interventions
J Med Internet Res 2011;13(4):e126
URL: http://www.jmir.org/2011/4/e126/

doi: 10.2196/jmir.1923
PMID: 22209829
* Required

Your name *
First Last

Your answer

Joan R. Cates

Primary Affiliation (short), City, Country *
University of Toronto, Toronto, Canada

Your answer

University of North Carolina at Chapel Hill

Your e-mail address *

joancates@unc.edu

Your answer

Title of your manuscript *
Provide the (draft) title of your manuscript.

Your answer

Evaluation of a Serious Video Game to Facilitate Conversations about Human Papillomavirus Vaccination for Preteens: Pilot Randomized Controlled Trial

Name of your App/Software/Intervention *
If there is a short and a long/alternate name, write the short name first and add the long name in brackets.

Your answer

Land of Secret Gardens [Protect Them]

Evaluated Version (if any)
e.g. "V1", "Release 2017-03-01", "Version 2.0.27913"

Your answer

N/A

Language(s) *
What language is the intervention/app in? If multiple languages are available, separate by comma (e.g. "English, French")

Your answer

English

URL of your Intervention Website or App
e.g. a direct link to the mobile app on app in appstore (itunes, Google Play), or URL of the website. If the intervention is a DVD or hardware, you can also link to an Amazon page.

Your answer

N/A

URL of an image/screenshot (optional)

Your answer

N/A

Accessibility *
Can an enduser access the intervention presently?

Yes - access is free and open

access only for special usergroups, not open

access is open to everyone, but requires payment/subscription/in-app purchases

app/intervention no longer accessible

Other:

Primary Medical Indication/Disease/Condition *
e.g. "Stress", "Diabetes", or define the target group in brackets after the condition, e.g. "Autism (Parents of children with)", "Alzheimers (Informal Caregivers of)"

Your answer

Human papillomavirus

Primary Outcomes measured in trial *
comma-separated list of primary outcomes reported in the trial

Your answer
HPV vaccination initiation and completion

Secondary/other outcomes

Are there any other outcomes the intervention is expected to affect?

Your answer
Knowledge of HPV and HPV vaccination, self efficacy and decisional balance, and game presence and game playing for intervention participants

Recommended "Dose" *
What do the instructions for users say on how often the app should be used?

Approximately Daily

Approximately Weekly

Approximately Monthly

Approximately Yearly

"as needed"

Other:

Preteens in the intervention group were asked to play the Land of Secret Gardens and complete 3 tasks in the video game. The tasks occurred in a sequence that required the player to return to the games multiple times. Participants were allotted up to 4 months to compete

Approx. Percentage of Users (starters) still using the app as recommended after 3 months *

Users after 3 months unknown / not evaluated

0-10%

11-20%

21-30%

31-40%

41-50%

51-60%

61-70%

71%-80%

81-90%

91-100%

Other:

Overall, was the app/intervention effective? *

yes: all primary outcomes were better (although non-significant in intervention group vs control)

partly: SOME primary outcomes were significantly better in intervention group vs control

For primary outcomes, there were no statistically significant difference between control and intervention

potentially harmful: control was significantly better than intervention in one or more outcomes

inconclusive: more research is needed

Other:

Article Preparation Status/Stage *
At which stage in your article preparation are you currently (at the time you fill in this form)

not submitted yet - in early draft status

not submitted yet - in late draft status, just before submission

submitted to a journal but not reviewed yet

submitted to a journal and after receiving initial reviewer comments

Yes - The article has been submitted to a journal and accepted, but not published yet

published

Other:

Journal *
If you already know where you will submit this paper (or if it is already submitted), please provide the journal name (if it is not JMIR, provide the journal name under "other")

not submitted yet / unclear where I will submit this

Journal of Medical Internet Research (JMIR)

JMIR mHealth and UHealth

Submission pending for JMIR Serious Games

JMIR Mental Health

JMIR Public Health

JMIR Formative Research

Other JMIR sister journal

Other:

Is this a full powered effectiveness trial or a pilot/feasibility trial? *

Pilot/feasibility (yes)

Fully powered

Cates JR, Fuemmeler BF, Diehl SJ, Stockton LL, Porter J, Ihekweazu Gurbani A and Coyne-Beasley T, Developing a Serious Videogame for Preteens to Motivate HPV Vaccination Decision Making: Land of Secret Gardens. Games for Health Journal, 2018. 7(1): p. 1-16.

Manuscript tracking number *
If this is a JMIR submission, please provide the manuscript tracking number under "other" (The ms tracking number can be found in the submission acknowledgement email, or when you login as author in JMIR. If the paper is already published in JMIR, then the ms tracking number is the four-digit number at the end of the DOI, to be found at the bottom of each published article in JMIR)

no ms number (yet) / not (yet) submitted to / published in JMIR

JMIR ms#16883

Other:

TITLE AND ABSTRACT

1a) TITLE: Identification as a randomized trial in the title

1a) Does your paper address CONSORT item 1a? *
I.e does the title contain the phrase "Randomized Controlled Trial"? (if not, explain the reason under "other")

Yes, the title includes randomized controlled trial

Other:

1a-i) Identify the mode of delivery in the title
Identify the mode of delivery. Preferably use “web-based” and/or “mobile” and/or “electronic game” in the title. Avoid ambiguous terms like “online”, “virtual”, “interactive”. Use “Internet-based” only if Intervention includes non-web-based Internet components (e.g. email), use “computer-based” or “electronic” only if offline products are used. Use “virtual” only in the context of “virtual reality” (3-D worlds). Use “online” only in the context of “online support groups”. Complement or substitute product names with broader terms for the class of products (such as “mobile” or “smart phone” instead of “iphone”), especially if the application runs on different platforms.

Evaluation of a serious video game

subitem not at all important
1
2
3
4
5 X

essential

Clear selection

Does your paper address subitem 1a-i? *
Copy and paste relevant sections from manuscript title (include quotes in quotation marks "like this" to indicate direct quotes from your manuscript), or elaborate on this item by providing additional information not in the ms, or briefly explain why the item is not applicable/relevant for your study

Your answer
Facilitate conversations about HPV vaccination

Conversations with preteens about HPV vaccinations

1a-ii) Non-web-based components or important co-interventions in title
Mention non-web-based components or important co-interventions in title, if any (e.g., “with telephone support”).

subitem not at all important
1
2
3
4
5X

essential

Clear selection

Does your paper address subitem 1a-ii?
Copy and paste relevant sections from manuscript title (include quotes in quotation marks "like this" to indicate direct quotes from your manuscript), or elaborate on this item by providing additional information not in the ms, or briefly explain why the item is not applicable/relevant for your study

Your answer
“Herein,we report on the evaluation of the Land of SecretGardens game. The aims of this pilot study are to evaluate (1)preliminary data to determine whether children who received the Land of Secret Gardens game had better self-reported outcomes related to HPV knowledge or vaccination self-efficacy compared with those in a control group who did not receive the game and (2) outcomes related to the game play experience(in-game autonomy and competence, presence in the game,intuitive controls, interest or enjoyment, and characteristics of playing the game) among those who received the game.”

1a-iii) Primary condition or target group in the title
Mention primary condition or target group in the title, if any (e.g., “for children with Type I Diabetes”) Example: A Web-based and Mobile Intervention with Telephone Support for Children with Type I Diabetes: Randomized Controlled Trial

subitem not at all important
1
2
3
4
5X

essential

Clear selection

Does your paper address subitem 1a-iii? *
Copy and paste relevant sections from manuscript title (include quotes in quotation marks "like this" to indicate direct quotes from your manuscript), or elaborate on this item by providing additional information not in the ms, or briefly explain why the item is not applicable/relevant for your study

Your answer

“Our approach has been to develop methods to address parent resistance and misunderstandings about why the vaccine is needed early in development before their children are sexually active [14,15].In addition, we have worked to develop strategies to address provider’s perceived barriers about discussing the vaccine with their patients (eg, helping them to develop talking points that can be used within a short patient visit) [16]. Finally,recognizing that serious video games may have the potential to educate preteens about sexually transmitted diseases or the utility of vaccination [17,18] and can be effective in promoting health behaviors in children and adolescents [19-23], we developed a serious video game to promote HPV vaccination among preteens [24].”

1b) ABSTRACT: Structured summary of trial design, methods, results, and conclusions
NPT extension: Description of experimental treatment, comparator, care providers, centers, and blinding status.

1b-i) Key features/functionalities/components of the intervention and comparator in the METHODS section of the ABSTRACT
Mention key features/functionalities/components of the intervention and comparator in the abstract. If possible, also mention theories and principles used for designing the site. Keep in mind the needs of systematic reviewers and indexers by including important synonyms. (Note: Only report in the abstract what the main paper is reporting. If this information is missing from the main body of text, consider adding it)

subitem not at all important
1
2
3
4
5X

essential

Clear selection

“Specifically, with input from preteens aged 11 to 12 years and their parents, we developed Land ofSecret Gardens, a serious video game designed to teach about vaccines through an immersive story and to motivate a decision to seek HPV vaccination [24].Putting all of these pieces together, we initiated the ProtectThem study, which was undertaken in 36 primary care practices with 97 providers (MD, DO, NP, and RN) in North Carolina.This was a multiple baseline study and included 3 waves of activity and adjustment in 2015, 2016, and 2017. The intervention was designed to promote communication among providers, parents, and preteens to increase HPV vaccination for preteens aged 11 to 12 years when the vaccine is most effective [25]. Communication tools for the providers included brochures, posters, web-based information for parents, and interactive web-based training for providers. In addition, as part of the intervention, we provided select patients access to theLand of the Secret Garden, an age-appropriate, entertaining web-based video game designed to educate preteens about HPVinfection and HPV vaccination and to promote conversations with parents and providers and the decision to vaccinate.”

Does your paper address subitem 1b-i? *
Copy and paste relevant sections from the manuscript abstract (include quotes in quotation marks "like this" to indicate direct quotes from your manuscript), or elaborate on this item by providing additional information not in the ms, or briefly explain why the item is not applicable/relevant for your study

Your answer

1b-ii) Level of human involvement in the METHODS section of the ABSTRACT

Clarify the level of human involvement in the abstract, e.g., use phrases like “fully automated” vs. “therapist/nurse/care provider/physician-assisted” (mention number and expertise of providers involved, if any). (Note: Only report in the abstract what the main paper is reporting. If this information is missing from the main body of text, consider adding it)

subitem not at all important
1
2
3
4
5X

essential

Clear selection

Does your paper address subitem 1b-ii?
Copy and paste relevant sections from the manuscript abstract (include quotes in quotation marks "like this" to indicate direct quotes from your manuscript), or elaborate on this item by providing additional information not in the ms, or briefly explain why the item is not applicable/relevant for your study

Your answer

“Vaccination against HPV is recommended for routine use in those aged 11 to 12 years in the United States, yet it is underutilized. We aimed to develop an educational video game to engage preteens in the decision to vaccinate.” “We screened 131 dyads of parents and preteens from 18 primary practices in North Carolina who had not initiated HPV vaccination.”

1b-iii) Open vs. closed, web-based (self-assessment) vs. face-to-face assessments in the METHODS section of the ABSTRACT
Mention how participants were recruited (online vs. offline), e.g., from an open access website or from a clinic or a closed online user group (closed usergroup trial), and clarify if this was a purely web-based trial, or there were face-to-face components (as part of the intervention or for assessment). Clearly say if outcomes were self-assessed through questionnaires (as common in web-based trials). Note: In traditional offline trials, an open trial (open-label trial) is a type of clinical trial in which both the researchers and participants know which treatment is being administered. To avoid confusion, use “blinded” or “unblinded” to indicated the level of blinding

instead of “open”, as “open” in web-based trials usually refers to “open access” (i.e. participants can self-enrol). (Note: Only report in the abstract what the main paper is reporting. If this information is missing from the main body of text, consider adding it)

subitem not at all important
1
2
3
4
5X

essential

Clear selection

Does your paper address subitem 1b-iii?
Copy and paste relevant sections from the manuscript abstract (include quotes in quotation marks "like this" to indicate direct quotes from your manuscript), or elaborate on this item by providing additional information not in the ms, or briefly explain why the item is not applicable/relevant for your study

Your answer

“Putting all of these pieces together, we initiated the Protect Them study, which was undertaken in 36 primary care practices with 97 providers (MD, DO, NP, and RN) in North Carolina. This was a multiple baseline study and included 3 waves of activity and adjustment in 2015, 2016, and 2017. The intervention was designed to promote communication among providers, parents, and preteens to increase HPV vaccination for preteens aged 11 to 12 years when the vaccine is most effective [25]. Communication tools for the providers included brochures, posters, web-based information for parents, and interactive web-based training for providers. In addition, as part of the intervention, we provided select patients access to the Land of the Secret Garden, an age-appropriate, entertaining web-based video game designed to educate preteens about HPV infection and HPV vaccination and to promote conversations with parents and providers and the decision to vaccinate.”

1b-iv) RESULTS section in abstract must contain use data
Report number of participants enrolled/assessed in each group, the use/uptake of the intervention (e.g., attrition/adherence metrics, use over time, number of logins etc.), in addition to primary/secondary outcomes. (Note: Only report in the abstract what the main paper is reporting. If this information is missing from the main body of text, consider adding it)

subitem not at all important
1
2
3
4
5X

essential

Clear selection

Does your paper address subitem 1b-iv?
Copy and paste relevant sections from the manuscript abstract (include quotes in quotation marks "like this" to indicate direct quotes from your manuscript), or elaborate on this item by providing additional information not in the ms, or briefly explain why the item is not applicable/relevant for your study

Your answer
“In total, 18 preteens reported playing the game. The vaccination self-efficacy score was higher in the comparison group than the intervention group (1.65 vs 1.45; P=.05). The overall mean decisional balance score trended toward greater support of vaccination, although differences between the groups were not significant.. Vaccine initiation and completion rates were higher in the intervention group (22% vs 15%; P=.31) than in the comparison group (9% vs 2%; P=.10), although the difference was not significant.”

1b-v) CONCLUSIONS/DISCUSSION in abstract for negative trials

Conclusions/Discussions in abstract for negative trials: Discuss the primary outcome - if the trial is negative (primary outcome not changed), and the intervention was not used, discuss whether negative results are attributable to lack of uptake and discuss reasons. (Note: Only report in the abstract what the main paper is reporting. If this information is missing from the main body of text, consider adding it)

N/A subitem not at all important
1
2
3
4
5

essential

Clear selection

Does your paper address subitem 1b-v?
Copy and paste relevant sections from the manuscript abstract (include quotes in quotation marks "like this" to indicate direct quotes from your manuscript), or elaborate on this item by providing additional information not in the ms, or briefly explain why the item is not applicable/relevant for your study

Your answer
N/A

INTRODUCTION

2a) In INTRODUCTION: Scientific background and explanation of rationale

2a-i) Problem and the type of system/solution
Describe the problem and the type of system/solution that is object of the study: intended as stand-alone intervention vs. incorporated in broader health care program? Intended for a

particular patient population? Goals of the intervention, e.g., being more cost-effective to other interventions, replace or complement other solutions? (Note: Details about the intervention are provided in “Methods” under 5)

subitem not at all important
1
2
3
4
5X

essential

Clear selection

Does your paper address subitem 2a-i? *
Copy and paste relevant sections from the manuscript (include quotes in quotation marks "like this" to indicate direct quotes from your manuscript), or elaborate on this item by providing additional information not in the ms, or briefly explain why the item is not applicable/relevant for your study

Your answer
“Although many interventions promoting HPV vaccination have focused on either the parent or provider separately with moderate success [6,7], it is increasingly being recognized that a multilevel approach may further broaden dissemination efforts [8,9]. Communication strategies have focused on giving accurate information about HPV vaccination and on training providers to give clear messages about the safety and efficacy of HPV vaccines [10].”

2a-ii) Scientific background, rationale: What is known about the (type of) system
Scientific background, rationale: What is known about the (type of) system that is the object of the study (be sure to discuss the use of similar systems for other conditions/diagnoses, if appropiate), motivation for the study, i.e. what are the reasons for and what is the context for this specific study, from which stakeholder viewpoint is the study performed, potential impact of findings [2]. Briefly justify the choice of the comparator.

subitem not at all important
1
2
3
4
5X

essential

Clear selection

Does your paper address subitem 2a-ii? *
Copy and paste relevant sections from the manuscript (include quotes in quotation marks "like this" to indicate direct quotes from your manuscript), or elaborate on this item by providing additional information not in the ms, or briefly explain why the item is not applicable/relevant for your study

Your answer
“In response to this challenge of suboptimal HPV vaccination, our interdisciplinary team (communication, public health, medicine, clinical psychology, biostatistics, health economics, and nursing) has been leading efforts to design and implement multilevel communication strategies that target parents, health care providers, and preteens [11-13]. For instance, our approach has been to develop methods to address parent resistance and misunderstandings about why the vaccine is needed early in development before their children are sexually active [14,15]. In addition, we have worked to develop strategies to address provider’s perceived barriers about discussing the vaccine with their patients (eg, helping them to develop talking points that can be used within a short patient visit) [16]. Finally, recognizing that serious video games may have the potential to educate preteens about sexually transmitted diseases or the utility of vaccination [17,18] and can be effective in promoting health behaviors in children and adolescents [19-23], we developed a serious video game to promote HPV vaccination among preteens [24]. Specifically, with input from preteens aged 11 to 12 years and their parents, we developed Land of Secret Gardens, a serious video game designed to teach about vaccines through an immersive story and to motivate a decision to seek HPV vaccination [24].”

2b) In INTRODUCTION: Specific objectives or hypotheses

Does your paper address CONSORT subitem 2b? *
Copy and paste relevant sections from the manuscript (include quotes in quotation marks "like this" to indicate direct quotes from your manuscript), or elaborate on this item by providing additional information not in the ms, or briefly explain why the item is not applicable/relevant for your study

Your answer

“ Our hypothesis was that raising awareness about HPV vaccination eases conversations about the vaccination. We created a story about a secret garden as a metaphor for a preteen’s body and keeping it healthy. The goal was to plant a lush secret garden and protect the seedlings by treating them with a potion when they sprout to keep them healthy as they mature. Points to buy seeds and create the potion were earned by playing minigames. The minigames included several versions of finding secret objects in a garden shed and another that involved shooting down spikey balls (ie, the HPV) before they land on budding plants. Throughout the play, players were exposed to messaging about HPV and the benefits of the vaccine [24]”

METHODS

3a) Description of trial design (such as parallel, factorial) including allocation ratio

Does your paper address CONSORT subitem 3a? *
Copy and paste relevant sections from the manuscript (include quotes in quotation marks "like this" to indicate direct quotes from your manuscript), or elaborate on this item by providing additional information not in the ms, or briefly explain why the item is not applicable/relevant for your study

Your answer
“Full HPV vaccination coverage has been challenging due, in part, to providers not making strong recommendations [1]. There also remain parental concerns about the vaccine. For instance, some parents perceived the risk of HPV infection to be negligible, expressed concern about side effects, and believed the vaccine might encourage promiscuous behavior or that it may be too costly [4,5]. Health care professionals have reported that these parental attitudes and concerns are barriers to vaccination [4]. Thus, understanding and addressing these barriers would be critical to target within the context of interventions designed to increase uptake.

Although many interventions promoting HPV vaccination have focused on either the parent or provider separately with moderate success [6,7], it is increasingly being recognized that a multilevel approach may further broaden dissemination efforts [8,9]. Communication strategies have focused on giving accurate information about HPV vaccination and on training providers to give clear messages about the safety and efficacy of HPV vaccines [10]"

3b) Important changes to methods after trial commencement (such as eligibility criteria), with reasons

Does your paper address CONSORT subitem 3b? *
Copy and paste relevant sections from the manuscript (include quotes in quotation marks "like this" to indicate direct quotes from your manuscript), or elaborate on this item by providing additional information not in the ms, or briefly explain why the item is not applicable/relevant for your study

Your answer
No changes in the protocol.

3b-i) Bug fixes, Downtimes, Content Changes
Bug fixes, Downtimes, Content Changes: ehealth systems are often dynamic systems. A description of changes to methods therefore also includes important changes made on the intervention or comparator during the trial (e.g., major bug fixes or changes in the functionality or content) (5-iii) and other "unexpected events" that may have influenced study design such as staff changes, system failures/downtimes, etc. [2].

subitem not at all important
1
2
3
4
5X

essential

Clear selection

Does your paper address subitem 3b-i?
Copy and paste relevant sections from the manuscript (include quotes in quotation marks "like this" to indicate direct quotes from your manuscript), or elaborate on this item by providing additional information not in the ms, or briefly explain why the item is not applicable/relevant for your study

Your answer
Due to challenges in recruitment, number of dyads increased from 10 per practice in wave 1 to 20 per practice in waves 2 and 3

4a) Eligibility criteria for participants

Does your paper address CONSORT subitem 4a? *
Copy and paste relevant sections from the manuscript (include quotes in quotation marks "like this" to indicate direct quotes from your manuscript), or elaborate on this item by providing additional information not in the ms, or briefly explain why the item is not applicable/relevant for your study

Your answer

No changes.

4a-i) Computer / Internet literacy
Computer / Internet literacy is often an implicit “de facto” eligibility criterion - this should be explicitly clarified.

subitem not at all important

1
2
3
4
5x

In addition to preteens who had not received any HPV vaccine, eligible dyads confirmed that they had access to the internet and a mobile device or personal computer to complete the surveys and play the video game.

essential

Clear selection

Does your paper address subitem 4a-i?
Copy and paste relevant sections from the manuscript (include quotes in quotation marks "like this" to indicate direct quotes from your manuscript), or elaborate on this item by providing additional information not in the ms, or briefly explain why the item is not applicable/relevant for your study

Your answer
Access to computer required for the study

4a-ii) Open vs. closed, web-based vs. face-to-face assessments:
Open vs. closed, web-based vs. face-to-face assessments: Mention how participants were recruited (online vs. offline), e.g., from an open access website or from a clinic, and clarify if this was a purely web-based trial, or there were face-to-face components (as part of the intervention or for assessment), i.e., to what degree got the study team to know the participant. In online-only trials, clarify if participants were quasi-anonymous and whether having multiple identities was possible or whether technical or logistical measures (e.g., cookies, email confirmation, phone calls) were used to detect/prevent these.

subitem not at all important
1

2
3
4
5X

essential

Clear selection

Does your paper address subitem 4a-ii? *
Copy and paste relevant sections from the manuscript (include quotes in quotation marks "like this" to indicate direct quotes from your manuscript), or elaborate on this item by providing additional information not in the ms, or briefly explain why the item is not applicable/relevant for your study

Your answer
“Participants of this video game evaluation study were part of the lager Protect Them study mentioned earlier in the Introduction section. Parent and preteen dyads were recruited by providers at 36 different clinical sites that included family medicine practices, pediatric practices, and health departments. To enroll in the study, clinical sites in the Protect Them study agreed to identify up to 10 parents (wave 1) of preteens aged 11 to 12 years and up to 20 parents (waves 2 and 3) of preteens. Preteens aged 11 to 12 years and who had not received any doses of HPV vaccine were eligible to participate in the study. Providers contacted potential dyads via letters or telephone after they identified eligible preteens from their electronic medical records. Interested dyads provided their contact information (ie, phone number and email address) to the research team and signed a Health Insurance Portability and Accountability Act (HIPAA) release of information form to allow the research team to determine their HPV vaccination status from their health care provider. A practice champion provided a copy of the HIPAA form and the parent contact information to project staff. The most common obstacle was a high proportion of clients with parents needing informed consent provided in Spanish, an accommodation not available for this study. After the names of eligible parents and preteens were passed to the research team, research staff invited parents to participate in a telephone conversation about informed consent with their preteen via email and telephone contacts. The study protocol required up to 3 attempts to reach parents by both email and telephone contact. Staff used an institutional review board (IRB)–approved script to conduct an informed consent procedure with the parent and then with the preteen if the parent provided consent. The script included a description of the intervention and the process of random assignment to a study group, explained the risks and benefits of participation, and reviewed the study activities and incentives for participation. Both parents and preteens were assured that their information was confidential and that participation was voluntary. In addition to preteens who had not received any HPV vaccine, eligible dyads confirmed that they had access to the internet and a mobile device or personal computer to complete the surveys and play the video game. They also provided a

mailing address to receive gift certificates. Once they were enrolled, dyads were randomly assigned in a 1:1 ratio to either the intervention group or control group using a simple randomization schedule generated by the study team's statistician. Those who were assigned to the intervention arm received the game, whereas those assigned to the control group arm did not receive the game. The study procedures were approved by the university's nonbiomedical IRB."

4a-iii) Information giving during recruitment
Information given during recruitment. Specify how participants were briefed for recruitment and in the informed consent procedures (e.g., publish the informed consent documentation as appendix, see also item X26), as this information may have an effect on user self-selection, user expectation and may also bias results.

subitem not at all important
1
2
3
4
5

essential

Clear selection

Does your paper address subitem 4a-iii?
Copy and paste relevant sections from the manuscript (include quotes in quotation marks "like this" to indicate direct quotes from your manuscript), or elaborate on this item by providing additional information not in the ms, or briefly explain why the item is not applicable/relevant for your study

Your answer
"All practices were asked to screen dyads as part of the intervention. Half of the practices

(18/36, 50%) screened at least one dyad, and a total of 131 dyads were referred to the research team. Of these 131 dyads, 16 (12.2%) did not meet the eligibility criteria for the study. Among the 115 that were eligible, 16 (13.9%) refused to participate in the study, and 42 (36.5%) dyads did not respond to repeated email and telephone contact. Almost half of the eligible dyads (55/115, 47.8%) completed the informed consent process and baseline surveys and were enrolled in the study, and 47 dyads completed the postsurvey."

4b) Settings and locations where the data were collected

Does your paper address CONSORT subitem 4b? *
Copy and paste relevant sections from the manuscript (include quotes in quotation marks "like this" to indicate direct quotes from your manuscript), or elaborate on this item by providing additional information not in the ms, or briefly explain why the item is not applicable/relevant for your study

Your answer
"A total of 36 primary care practices across 3 regions in North Carolina enrolled in the Protect Them study (Figure 1)—12 practices were recruited from the eastern region during wave 1 (February 2015 to June 2015), 18 practices were recruited from the central region during wave 2 (February 2016 to July 2016), and 6 practices were recruited from the western region during wave 3 (February 2017 to October 2017)"

4b-i) Report if outcomes were (self-)assessed through online questionnaires
Clearly report if outcomes were (self-)assessed through online questionnaires (as common in web-based trials) or otherwise.

subitem not at all important
1
2
3
4
5X

essential

Clear selection

Does your paper

Copy and paste relevant sections from the manuscript (include quotes in quotation marks "like this" to indicate direct quotes from your manuscript), or elaborate on this item by providing additional information not in the ms, or briefly explain why the item is not applicable/relevant for your study

Your answer

“We asked all parents and preteens to complete the baseline and post intervention surveys. The surveys were designed in Qualtrics [29], and survey links were sent to the parent’s email address. Dyads received the baseline surveys before potential exposure to the video game and parent portal. Up to 5 email reminders and 3 telephone calls were made to encourage survey completion. Follow-up reminders were offered to encourage game play and completion of the task. Each participant received a US $25 gift certificate to Walmart if they completed a survey subitem 4b-i? *

4b-ii) Report how institutional affiliations are displayed
Report how institutional affiliations are displayed to potential participants [on ehealth media], as affiliations with prestigious hospitals or universities may affect volunteer rates, use, and reactions with regards to an intervention.(Not a required item – describe only if this may bias results)

subitem not at all important
1
2
3
4
5X

essential

Clear selection

Does your paper address subitem 4b-ii?
Copy and paste relevant sections from the manuscript (include quotes in quotation marks "like this" to indicate direct quotes from your manuscript), or elaborate on this item by providing additional information not in the ms, or briefly explain why the item is not applicable/relevant for your study

Your answer
Institutional affiliations have not been relevant. Study sponsored by the University of North Carolina at Chapel Hill.

5) The interventions for each group with sufficient details to allow replication, including how and when they were actually administered. Study groups were asked identical questions about their knowledge and attitudes about HPV and their intentions to vaccinate against HPV at baseline. Post intervention surveys were sent 4 months after participants completed their baseline surveys. All participants were asked if the preteen received any dose of the HPV vaccine. Participants in the intervention group were asked additional questions about their experience with the intervention and the Protect Them resources.

5-i) Mention names, credential, affiliations of the developers, sponsors, and owners
Mention names, credential, affiliations of the developers, sponsors, and owners [6] (if authors/evaluators are owners or developer of the software, this needs to be declared in a “Conflict of interest” section or mentioned elsewhere in the manuscript).

subitem not at all important
1
2
3
4
5X

essential

Clear selection

Sponsor is NIH NIAID,

Does your paper address subitem 5-i?
Copy and paste relevant sections from the manuscript (include quotes in quotation marks "like this" to indicate direct quotes from your manuscript), or elaborate on this item by providing additional information not in the ms, or briefly explain why the item is not applicable/relevant for your study

Your answer

Information in manuscript is endorsed collectively.

5-ii) Describe the history/development process
Describe the history/development process of the application and previous formative evaluations (e.g., focus groups, usability testing), as these will have an impact on adoption/use rates and help with interpreting results.

Formative research is collectively reported here:

subitem not at all important
1
2
3
4
5X

essential

Clear selection

Does your paper address subitem 5-ii?
Copy and paste relevant sections from the manuscript (include quotes in quotation marks "like this" to indicate direct quotes from your manuscript), or elaborate on this item by providing additional information not in the ms, or briefly explain why the item is not applicable/relevant for your study

Your answer

Formative research on Land of Secret Gardens is reported here;

Cates JR, Fuemmeler BF, Diehl SJ, Stockton LL, Porter J, Ihekweazu C, et al. Developing a serious videogame for preteens to motivate HPV vaccination decision making: land of secret gardens. Games Health J 2018 Feb;7(1):51-66 [FREE Full text] [doi: 10.1089/g4h.2017.0002] [Medline: 29161529]

5-iii) Revisions and updating
Revisions and updating. Clearly mention the date and/or version number of the application/intervention (and comparator, if applicable) evaluated, or describe whether the intervention underwent major changes during the evaluation process, or whether the development and/or content was “frozen” during the trial. Describe dynamic components such as news feeds or changing content which may have an impact on the replicability of the intervention (for unexpected events see item 3b).

subitem not at all important
1
2
3
4
5

essential

Clear selection

Does your paper address subitem 5-iii?
Copy and paste relevant sections from the manuscript (include quotes in quotation marks "like this" to indicate direct quotes from your manuscript), or elaborate on this item by providing additional information not in the ms, or briefly explain why the item is not applicable/relevant for your study

Your answer
The intervention did not undergo major changes during the evaluation process.

5-iv) Quality assurance methods
Provide information on quality assurance methods to ensure accuracy and quality of information provided [1], if applicable.

subitem not at all important
1
2
3
4
5

essential

Clear selection

Does your paper address subitem 5-iv?
Copy and paste relevant sections from the manuscript (include quotes in quotation marks "like this" to indicate direct quotes from your manuscript), or elaborate on this item by providing additional information not in the ms, or briefly explain why the item is not applicable/relevant for your study

Your answer
“Preteens in the intervention group were asked to play the Land of Secret Gardens and complete 3 tasks in the video game. The tasks occurred in a sequence that required the player to return to the game multiple times. A badge appeared on the leaderboard on completion of each task, which allowed preteens to track their progress. Preteens were exposed to messages about HPV and the HPV vaccination throughout the game, and continued use of the game would result in greater message exposure. Project staff monitored the game play progress for each participant with Navicat [30] and sent reminders to parents to encourage game play. Instructions for parents to guide game play were posted on the parent portal, along with a video

that described the background and premise of the game. A help form was also available on the parent portal to request technical support for the game.”

5-v) Ensure replicability by publishing the source code, and/or providing screenshots/screen-capture video, and/or providing flowcharts of the algorithms used
Ensure replicability by publishing the source code, and/or providing screenshots/screen-capture video, and/or providing flowcharts of the algorithms used. Replicability (i.e., other researchers should in principle be able to replicate the study) is a hallmark of scientific reporting.

subitem not at all important
1
2
3
4
5

essential

Clear selection

Does your paper address subitem 5-v?
Copy and paste relevant sections from the manuscript (include quotes in quotation marks "like this" to indicate direct quotes from your manuscript), or elaborate on this item by providing additional information not in the ms, or briefly explain why the item is not applicable/relevant for your study

Your answer

Screen shots in

Multimedia Appendix 1 Video game for preteens. [PPTX File , 7094 KB-Multimedia Appendix 1]

Cates JR, Fuemmeler BF, Diehl SJ, Stockton LL, Porter J, Ihekweazu C, et al. Developing a serious videogame for preteens to motivate HPV vaccination decision making: land of secret gardens. Games Health J 2018 Feb;7(1):51-66 [FREE Full text] [doi: 10.1089/g4h.2017.0002] [Medline: 29161529]

5-vi) Digital preservation
Digital preservation: Provide the URL of the application, but as the intervention is likely to change or disappear over the course of the years; also make sure the intervention is archived (Internet Archive, webcitation.org, and/or publishing the source code or screenshots/videos alongside the article). As pages behind login screens cannot be archived, consider creating demo pages which are accessible without login.

subitem not at all important
1
2
3
4
5X

essential

Clear selection

Does your paper address subitem 5-vi?
Copy and paste relevant sections from the manuscript (include quotes in quotation marks "like this" to indicate direct quotes from your manuscript), or elaborate on this item by providing additional information not in the ms, or briefly explain why the item is not applicable/relevant for your study

Your answer

Screen shots in appendix:

Cates JR, Fuemmeler BF, Diehl SJ, Stockton LL, Porter J, Ihekweazu C, et al. Developing a serious videogame for preteens to motivate HPV vaccination decision making: land of secret gardens. Games Health J 2018 Feb;7(1):51-66 [FREE Full text] [doi: 10.1089/g4h.2017.0002] [Medline: 29161529]

5-vii) Access
Access: Describe how participants accessed the application, in what setting/context, if they had to pay (or were paid) or not, whether they had to be a member of specific group. If known, describe how participants obtained “access to the platform and Internet” [1]. To ensure access for editors/reviewers/readers, consider to provide a “backdoor” login account or demo mode for reviewers/readers to explore the application (also important for archiving purposes, see vi).

subitem not at all important
1
2
3
4
5X

essential

Clear selection

Does your paper address subitem 5-vii? *
Copy and paste relevant sections from the manuscript (include quotes in quotation marks "like this" to indicate direct quotes from your manuscript), or elaborate on this item by providing additional information not in the ms, or briefly explain why the item is not applicable/relevant for your study

Your answer

Our paper is available via open access.

5-viii) Mode of delivery, features/functionalities/components of the intervention and comparator, and the theoretical framework
Describe mode of delivery, features/functionalities/components of the intervention and comparator, and the theoretical framework [6] used to design them (instructional strategy [1],

behaviour change techniques, persuasive features, etc., see e.g., [7, 8] for terminology). This includes an in-depth description of the content (including where it is coming from and who developed it) [1],” whether [and how] it is tailored to individual circumstances and allows users to track their progress and receive feedback” [6]. This also includes a description of communication delivery channels and – if computer-mediated communication is a component – whether communication was synchronous or asynchronous [6]. It also includes information on presentation strategies [1], including page design principles, average amount of text on pages, presence of hyperlinks to other resources, etc. [1].

subitem not at all important
1
2
3
4
5X

essential

Clear selection

Does your paper address subitem 5-viii? *
Copy and paste relevant sections from the manuscript (include quotes in quotation marks "like this" to indicate direct quotes from your manuscript), or elaborate on this item by providing additional information not in the ms, or briefly explain why the item is not applicable/relevant for your study

Your answer

“The video game incorporated gamification elements (eg, points, badges for completion of tasks, and a leaderboard) [26] to increase interest among players while also aiming to increase preteen knowledge and vaccine self-efficacy (ie, confidence in getting the vaccine despite barriers). Self-determination theory [27] was also used to inform the game design, as this has previously been used to evaluate the motivational pull of video games [23]. In addition, the game included an immersive story to enhance motivation to play the game [23,28] and engender deeper information processing [21]. Our hypothesis was that raising awareness about HPV vaccination eases conversations about the vaccination. We created a story about a secret

garden as a metaphor for a preteen’s body and keeping it healthy. The goal was to plant a lush secret garden and protect the seedlings by treating them with a potion when they sprout to keep them healthy as they mature. Points to buy seeds and create the potion were earned by playing minigames. The minigames included several versions of finding secret objects in a garden shed and another that involved shooting down spikey balls (ie, the HPV) before they land on budding plants. Throughout the play, players were exposed to messaging about HPV and the benefits of the vaccine [24].”

5-ix) Describe use parameters
Describe use parameters (e.g., intended “doses” and optimal timing for use). Clarify what instructions or recommendations were given to the user, e.g., regarding timing, frequency, heaviness of use, if any, or was the intervention used ad libitum.

subitem not at all important
1
2
3
4
5X

essential

Clear selection

Does your paper address subitem 5-ix?
Copy and paste relevant sections from the manuscript (include quotes in quotation marks "like this" to indicate direct quotes from your manuscript), or elaborate on this item by providing additional information not in the ms, or briefly explain why the item is not applicable/relevant for your study

Your answer

“The study protocol required up to 3 attempts to reach parents by both email and telephone contact. Staff used an institutional review board (IRB)–approved script to conduct an informed consent procedure with the parent and then with the preteen if the parent provided consent. The

script included a description of the intervention and the process of random assignment to a study group, explained the risks and benefits of participation, and reviewed the study activities and incentives for participation. Both parents and preteens were assured that their information was confidential and that participation was voluntary. In addition to preteens who had not received any HPV vaccine, eligible dyads confirmed that they had access to the internet and a mobile device or personal computer to complete the surveys and play the video game. They also provided a mailing address to receive gift certificates. Once they were enrolled, dyads were randomly assigned in a 1:1 ratio to either the intervention group or control group using a simple randomization schedule generated by the study team's statistician. Those who were assigned to the intervention arm received the game, whereas those assigned to the control group arm did not receive the game. The study procedures were approved by the university's nonbiomedical IRB."

5-x) Clarify the level of human involvement
Clarify the level of human involvement (care providers or health professionals, also technical assistance) in the e-intervention or as co-intervention (detail number and expertise of professionals involved, if any, as well as "type of assistance offered, the timing and frequency of the support, how it is initiated, and the medium by which the assistance is delivered". It may be necessary to distinguish between the level of human involvement required for the trial, and the level of human involvement required for a routine application outside of a RCT setting (discuss under item 21 – generalizability).

subitem not at all important
1
2
3
4X
5

essential

Clear selection

Does your paper address subitem 5-x?
Copy and paste relevant sections from the manuscript (include quotes in quotation marks "like this" to indicate direct quotes from your manuscript), or elaborate on this item by providing

additional information not in the ms, or briefly explain why the item is not applicable/relevant for your study

Your answer

“Parent and preteen dyads were recruited by providers at 36 different clinical sites that included family medicine practices, pediatric practices, and health departments. To enroll in the study, clinical sites in the Protect Them study agreed to identify up to 10 parents (wave 1) of preteens aged 11 to 12 years and up to 20 parents (waves 2 and 3) of preteens. Preteens aged 11 to 12 years and who had not received any doses of HPV vaccine were eligible to participate in the study. Providers contacted potential dyads via letters or telephone after they identified eligible preteens from their electronic medical records. Interested dyads provided their contact information (ie, phone number and email address) to the research team and signed a Health Insurance Portability and Accountability Act (HIPAA) release of information form to allow the research team to determine their HPV vaccination status from their health care provider. A practice champion provided a copy of the HIPAA form and the parent contact information to project staff. The most common obstacle was a high proportion of clients with parents needing informed consent provided in Spanish, an accommodation not available for this study.”

5-xi) Report any prompts/reminders used
Report any prompts/reminders used: Clarify if there were prompts (letters, emails, phone calls, SMS) to use the application, what triggered them, frequency etc. It may be necessary to distinguish between the level of prompts/reminders required for the trial, and the level of prompts/reminders for a routine application outside of a RCT setting (discuss under item 21 – generalizability).

subitem not at all important
1
2
3
4
5

essential

Clear selection

Does your paper address subitem 5-xi? *
Copy and paste relevant sections from the manuscript (include quotes in quotation marks "like this" to indicate direct quotes from your manuscript), or elaborate on this item by providing additional information not in the ms, or briefly explain why the item is not applicable/relevant for your study

Your answer
“After the names of eligible parents and preteens were passed to the research team, research staff invited parents to participate in a telephone conversation about informed consent with their preteen via email and telephone contacts. The study protocol required up to 3 attempts to reach parents by both email and telephone contact. Staff used an institutional review board (IRB)–approved script to conduct an informed consent procedure with the parent and then with the preteen if the parent provided consent. The script included a description of the intervention and the process of random assignment to a study group, explained the risks and benefits of participation, and reviewed the study activities and incentives for participation. Both parents and preteens were assured that their information was confidential and that participation was voluntary.”

5-xii) Describe any co-interventions (incl. training/support)
Describe any co-interventions (incl. training/support): Clearly state any interventions that are provided in addition to the targeted eHealth intervention, as ehealth intervention may not be designed as stand-alone intervention. This includes training sessions and support [1]. It may be necessary to distinguish between the level of training required for the trial, and the level of training for a routine application outside of a RCT setting (discuss under item 21 – generalizability.

subitem not at all important
1
2
3
4
5X

essential

Clear selection

Does your paper address subitem 5-xii? *

Copy and paste relevant sections from the manuscript (include quotes in quotation marks "like this" to indicate direct quotes from your manuscript), or elaborate on this item by providing additional information not in the ms, or briefly explain why the item is not applicable/relevant for your study

Your answer

“Putting all of these pieces together, we initiated the Protect Them study, which was undertaken in 36 primary care practices with 97 providers (MD, DO, NP, and RN) in North Carolina. This was a multiple baseline study and included 3 waves of activity and adjustment in 2015, 2016, and 2017. The intervention was designed to promote communication among providers, parents, and preteens to increase HPV vaccination for preteens aged 11 to 12 years when the vaccine is most effective [25]. Communication tools for the providers included brochures, posters, web-based information for parents, and interactive web-based training for providers. In addition, as part of the intervention, we provided select patients access to the Land of the Secret Garden, an age-appropriate, entertaining web-based video game designed to educate preteens about HPV infection and HPV vaccination and to promote conversations with parents and providers and the decision to vaccinate.”

6a) Completely defined pre-specified primary and secondary outcome measures, including how and when they were assessed

Does your paper address CONSORT subitem 6a? *

Copy and paste relevant sections from the manuscript (include quotes in quotation marks "like this" to indicate direct quotes from your manuscript), or elaborate on this item by providing additional information not in the ms, or briefly explain why the item is not applicable/relevant for your study

Your answer

We assigned preteens in the intervention group to play the video game and asked their parents to review web-based materials and then collected baseline and post intervention data from both study groups via Qualtrics surveys. From the practice champion, we collected primary outcomes

of initiations and completion of the HPV vaccination status of each preteen participant approximately 9 months post intervention. For secondary outcomes, we measured HPV and HPV vaccine knowledge, HPV vaccination self-efficacy, and HPV decisional balance in both intervention and comparison groups. From the intervention group only, we collected data on Physical/Emotional/Narrative Presence Scale (PENS) [31] to gauge preteens' immersion in the game and game play characteristics.

6a-i) Online questionnaires: describe if they were validated for online use and apply CHERRIES items to describe how the questionnaires were designed/deployed
If outcomes were obtained through online questionnaires, describe if they were validated for online use and apply CHERRIES items to describe how the questionnaires were designed/deployed [9].

subitem not at all important
1
2
3
4X
5

essential

Clear selection

Does your paper address subitem 6a-i?
Copy and paste relevant sections from manuscript text

Your answer

The online questionnaires had been validated with older age groups and adapted for our preteen participants (self efficacy and decisional balance scales). We did not use CHERRIES items.

6a-ii) Describe whether and how “use” (including intensity of use/dosage) was defined/measured/monitored

Describe whether and how “use” (including intensity of use/dosage) was defined/measured/monitored (logins, logfile analysis, etc.). Use/adoption metrics are important process outcomes that should be reported in any ehealth trial.

subitem not at all important

1

2

3

4X

5

essential

Clear selection

Does your paper address subitem 6a-ii?

Copy and paste relevant sections from manuscript text

Your answer

“We assigned preteens in the intervention group to play the video game and asked their parents to review web-based materials and then collected baseline and postintervention data from both study groups via Qualtrics surveys. We measured knowledge, vaccination self-efficacy, and decisional balance in both groups. From the intervention group only, we collected data on Physical/Emotional/Narrative Presence Scale (PENS) [31] to gauge preteens’ immersion in the game. Finally, from the practice champion, we collected the HPV vaccination status of each preteen participant approximately 9 months postintervention. The description of each measure is as follows: • Knowledge scale: asked in both the intervention and comparison groups. The 5 items asked whether HPV vaccination (1) can prevent genital warts, (2) can prevent cervical cancer, (3) can prevent anal cancer, (4) can prevent throat cancer, and (5) is recommended for 11- and 12-year-old boys and girls. • Vaccination Self-efficacy and decisional balance scales [32]: used in the intervention group and the comparison group to compare self-efficacy and intentions to vaccinate and decisional balance. We used 8 items for vaccination self-efficacy, 4

items for positive decision to vaccinate, and 5 items for negative decision to vaccinate. All questions were rated on a 3-point Likert scale (1=not at all, 2=somewhat, and 3=a lot). • PENS [31]: to learn more about the intervention experience, we collected measures specific to preteens in the intervention group. We used measurements from PENS to gauge the extent of the preteen's immersion in the story. • Game play: we asked participants who played the game how they played the game, for example, earning at least one shield, playing 3 or more times, playing more than 10 min per session, playing with a parent or sibling, playing the shield game and the hidden objects game, creating a potion, and correctly identifying the game metaphor. In addition, we recruited 3 postintervention focus groups with preteens (2 or 3 in each group) who played the game and asked about their experience. The focus groups were conducted and recorded via telephone calls. Parents gave consent before the preteens joined the conversation. The study moderator asked whether the preteens enjoyed the game and which parts they enjoyed or did not enjoy. • HPV immunization records: obtained for all preteen participants from practice champions approximately 9 months following each intervention. This period allowed enough interval for the preteens to complete their HPV vaccination series. Statistical Analysis We compared postintervention knowledge"

6a-iii) Describe whether, how, and when qualitative feedback from participants was obtained
Describe whether, how, and when qualitative feedback from participants was obtained (e.g., through emails, feedback forms, interviews, focus groups).

subitem not at all important
1
2
3
4
5

essential

Clear selection

Does your paper address subitem 6a-iii?
Copy and paste relevant sections from manuscript text

Your answer
“In addition, we recruited 3 postintervention focus groups with preteens (2 or 3 in each group) who played the game and asked about their experience. The focus groups were conducted and recorded via telephone calls. Parents gave consent before the preteens joined the conversation. The study moderator asked whether the preteens enjoyed the game and which parts they enjoyed or did not enjoy.”

6b) Any changes to trial outcomes after the trial commenced, with reasons

Does your paper address CONSORT subitem 6b? *
Copy and paste relevant sections from the manuscript (include quotes in quotation marks "like this" to indicate direct quotes from your manuscript), or elaborate on this item by providing additional information not in the ms, or briefly explain why the item is not applicable/relevant for your study

Your answer
No changes to the trial outcomes.

7a) How sample size was determined
NPT: When applicable, details of whether and how the clustering by care provides or centers was addressed

7a-i) Describe whether and how expected attrition was taken into account when calculating the sample size
Describe whether and how expected attrition was taken into account when calculating the sample size.

subitem not at all important
1
2
3
4
5X

essential

Clear selection

Does your paper address subitem 7a-i?
Copy and paste relevant sections from manuscript title (include quotes in quotation marks "like this" to indicate direct quotes from your manuscript), or elaborate on this item by providing additional information not in the ms, or briefly explain why the item is not applicable/relevant for your study

Your answer
“After the names of eligible parents and preteens were passed to the research team, research staff invited parents to participate in a telephone conversation about informed consent with their preteen via email and telephone contacts. The study protocol required up to 3 attempts to reach parents by both email and telephone contact. Staff used an institutional review board (IRB)–approved script to conduct an informed consent procedure with the parent and then with the preteen if the parent provided consent. The script included a description of the intervention and the process of random assignment to a study group, explained the risks and benefits of participation, and reviewed the study activities and incentives for participation. Both parents and preteens were assured that their information was confidential and that participation was voluntary. In addition to preteens who had not received any HPV vaccine, eligible dyads confirmed that they had access to the internet and a mobile device or personal computer to complete the surveys and play the video game. They also provided a mailing address to receive gift certificates. Once they were enrolled, dyads were randomly assigned in a 1:1 ratio to either the intervention group or control group using a simple randomization schedule generated by the study team’s statistician. Those who were assigned to the intervention arm received the game, whereas those assigned to the control group arm did not receive the game. The study procedures were approved by the university’s nonbiomedical IRB.”

7b) When applicable, explanation of any interim analyses and stopping guidelines

Does your paper address CONSORT subitem 7b? *
Copy and paste relevant sections from the manuscript (include quotes in quotation marks "like this" to indicate direct quotes from your manuscript), or elaborate on this item by providing additional information not in the ms, or briefly explain why the item is not applicable/relevant for your study

Your answer

“Parent and preteen dyads were recruited by providers at 36 different clinical sites that included family medicine practices, pediatric practices, and health departments. To enroll in the study, clinical sites in the Protect Them study agreed to identify up to 10 parents (wave 1) of preteens aged 11 to 12 years and up to 20 parents (waves 2 and 3) of preteens. Preteens aged 11 to 12 years and who had not received any doses of HPV vaccine were eligible to participate in the study. Providers contacted potential dyads via letters or telephone after they identified eligible preteens from their electronic medical records. Interested dyads provided their contact information (ie, phone number and email address) to the research team and signed a Health Insurance Portability and Accountability Act (HIPAA) release of information form to allow the research team to determine their HPV vaccination status from their health care provider. A practice champion provided a copy of the HIPAA form and the parent contact information to project staff.”

8a) Method used to generate the random allocation sequence
NPT: When applicable, how care providers were allocated to each trial group

Does your paper address CONSORT subitem 8a? *
Copy and paste relevant sections from the manuscript (include quotes in quotation marks "like this" to indicate direct quotes from your manuscript), or elaborate on this item by providing additional information not in the ms, or briefly explain why the item is not applicable/relevant for your study

Your answer

“Once they were enrolled, dyads were randomly assigned in a 1:1 ratio to either the intervention group or control group using a simple randomization schedule generated by the study team’s statistician. Those who were assigned to the intervention arm received the game, whereas those assigned to the control group arm did not receive the game. The study procedures were approved by the university’s nonbiomedical IRB.”

8b) Type of randomisation; details of any restriction (such as blocking and block size)

Does your paper address CONSORT subitem 8b? *

Copy and paste relevant sections from the manuscript (include quotes in quotation marks "like this" to indicate direct quotes from your manuscript), or elaborate on this item by providing additional information not in the ms, or briefly explain why the item is not applicable/relevant for your study

Your answer
“After the names of eligible parents and preteens were passed to the research team, research staff invited parents to participate in a telephone conversation about informed consent with their preteen via email and telephone contacts. The study protocol required up to 3 attempts to reach parents by both email and telephone contact. Staff used an institutional review board (IRB)–approved script to conduct an informed consent procedure with the parent and then with the preteen if the parent provided consent. The script included a description of the intervention and the process of random assignment to a study group, explained the risks and benefits of participation, and reviewed the study activities and incentives for participation. Both parents and preteens were assured that their information was confidential and that participation was voluntary. In addition to preteens who had not received any HPV vaccine, eligible dyads confirmed that they had access to the internet and a mobile device or personal computer to complete the surveys and play the video game. They also provided a mailing address to receive gift certificates.”

9) Mechanism used to implement the random allocation sequence (such as sequentially numbered containers), describing any steps taken to conceal the sequence until interventions were assigned

Does your paper address CONSORT subitem 9? *
Copy and paste relevant sections from the manuscript (include quotes in quotation marks "like this" to indicate direct quotes from your manuscript), or elaborate on this item by providing additional information not in the ms, or briefly explain why the item is not applicable/relevant for your study

Your answer
No additional steps were used.

10) Who generated the random allocation sequence, who enrolled participants, and who assigned participants to interventions

Does your paper address CONSORT subitem 10? *
Copy and paste relevant sections from the manuscript (include quotes in quotation marks "like this" to indicate direct quotes from your manuscript), or elaborate on this item by providing additional information not in the ms, or briefly explain why the item is not applicable/relevant for your study

Your answer
Once they were enrolled, dyads were randomly assigned in a 1:1 ratio to either the intervention group or control group using a simple randomization schedule generated by the study team's statistician. Those who were assigned to the intervention arm received the game, whereas those assigned to the control group arm did not receive the game.

11a) If done, who was blinded after assignment to interventions (for example, participants, care providers, those assessing outcomes) and how
NPT: Whether or not administering co-interventions were blinded to group assignment

11a-i) Specify who was blinded, and who wasn't
Specify who was blinded, and who wasn't. Usually, in web-based trials it is not possible to blind the participants [1, 3] (this should be clearly acknowledged), but it may be possible to blind outcome assessors, those doing data analysis or those administering co-interventions (if any).

subitem not at all important
1
2
3
4
5

essential

Clear selection

Does your paper address subitem 11a-i? *
Copy and paste relevant sections from the manuscript (include quotes in quotation marks "like this" to indicate direct quotes from your manuscript), or elaborate on this item by providing additional information not in the ms, or briefly explain why the item is not applicable/relevant for your study

Your answer
Participants were not blinded.

11a-ii) Discuss e.g., whether participants knew which intervention was the “intervention of interest” and which one was the “comparator”
Informed consent procedures (4a-ii) can create biases and certain expectations - discuss e.g., whether participants knew which intervention was the “intervention of interest” and which one was the “comparator”.

subitem not at all important
1
2
3
4
5

essential

Clear selection

Does your paper address subitem 11a-ii?
Copy and paste relevant sections from the manuscript (include quotes in quotation marks "like this" to indicate direct quotes from your manuscript), or elaborate on this item by providing additional information not in the ms, or briefly explain why the item is not applicable/relevant for your study

Your answer
Participants either received the intervention or not.

11b) If relevant, description of the similarity of interventions
(this item is usually not relevant for ehealth trials as it refers to similarity of a placebo or sham intervention to a active medication/intervention)

Does your paper address CONSORT subitem 11b? *
Copy and paste relevant sections from the manuscript (include quotes in quotation marks "like this" to indicate direct quotes from your manuscript), or elaborate on this item by providing additional information not in the ms, or briefly explain why the item is not applicable/relevant for your study

Your answer
N/A

12a) Statistical methods used to compare groups for primary and secondary outcomes
NPT: When applicable, details of whether and how the clustering by care providers or centers was addressed

Does your paper address CONSORT subitem 12a? *
Copy and paste relevant sections from the manuscript (include quotes in quotation marks "like this" to indicate direct quotes from your manuscript), or elaborate on this item by providing additional information not in the ms, or briefly explain why the item is not applicable/relevant for your study

Your answer
“We compared postintervention knowledge, vaccination self-efficacy, and decisional balance in the intervention and comparison groups using two-sample t tests, with significance level of α=.05. Immunization records were obtained for all preteen participants approximately 9 months postintervention for each cohort total. During the time of the study, the HPV vaccine was offered at both a 2-dose schedule with up to 6 to 12 months after the first shot and a 3-dose schedule with up to 6 months after the first shot. Practice champions were asked whether a preteen was

on a 2- or 3-dose HPV vaccination schedule and to confirm whether they initiated and/or completed the vaccine series. The intervention and comparison groups were compared regarding the initiation and completion of the vaccine series using the Mantel-Haenszel chi-square test stratified by intervention wave.”

12a-i) Imputation techniques to deal with attrition / missing values
Imputation techniques to deal with attrition / missing values: Not all participants will use the intervention/comparator as intended and attrition is typically high in ehealth trials. Specify how participants who did not use the application or dropped out from the trial were treated in the statistical analysis (a complete case analysis is strongly discouraged, and simple imputation techniques such as LOCF may also be problematic [4]).

subitem not at all important
1
2
3
4X
5

essential

Clear selection

Does your paper address subitem 12a-i? *
Copy and paste relevant sections from the manuscript (include quotes in quotation marks "like this" to indicate direct quotes from your manuscript), or elaborate on this item by providing additional information not in the ms, or briefly explain why the item is not applicable/relevant for your study

Your answer

“All practices were asked to screen dyads as part of the intervention. Half of the practices (18/36, 50%) screened at least one dyad, and a total of 131 dyads were referred to the research

team. Of these 131 dyads, 16 (12.2%) did not meet the eligibility criteria for the study. Among the 115 that were eligible, 16 (13.9%) refused to participate in the study, and 42 (36.5%) dyads did not respond to repeated email and telephone contact. Almost half of the eligible dyads (55/115, 47.8%) completed the informed consent process and baseline surveys and were enrolled in the study, and 47 dyads completed the postsurvey. The final study sample available for this evaluation of the game consisted of 28 preteens in the intervention group and 27 in the comparison group (21 and 26, respectively, completed the study)."

12b) Methods for additional analyses, such as subgroup analyses and adjusted analyses

Does your paper address CONSORT subitem 12b? *

Copy and paste relevant sections from the manuscript (include quotes in quotation marks "like this" to indicate direct quotes from your manuscript), or elaborate on this item by providing additional information not in the ms, or briefly explain why the item is not applicable/relevant for your study

Your answer

"We compared postintervention knowledge, vaccination self-efficacy, and decisional balance in the intervention and comparison groups using two-sample t tests, with significance level of α=.05. Immunization records were obtained for all preteen participants approximately 9 months postintervention for each cohort total. During the time of the study, the HPV vaccine was offered at both a 2-dose schedule with up to 6 to 12 months after the first shot and a 3-dose schedule with up to 6 months after the first shot. Practice champions were asked whether a preteen was on a 2- or 3-dose HPV vaccination schedule and to confirm whether they initiated and/or completed the vaccine series. The intervention and comparison groups were compared regarding the initiation and completion of the vaccine series using the Mantel-Haenszel chi-square test stratified by intervention wave."

X26) REB/IRB Approval and Ethical Considerations [recommended as subheading under "Methods"] (not a CONSORT item)

X26-i) Comment on ethics committee approval

subitem not at all important
1
2
3
4
5X

essential

Clear selection

Does your paper address subitem X26-i?
Copy and paste relevant sections from the manuscript (include quotes in quotation marks "like this" to indicate direct quotes from your manuscript), or elaborate on this item by providing additional information not in the ms, or briefly explain why the item is not applicable/relevant for your study

Your answer

Approved by UNC Biomedical IRB.

x26-ii) Outline informed consent procedures
Outline informed consent procedures e.g., if consent was obtained offline or online (how? Checkbox, etc.?), and what information was provided (see 4a-ii). See [6] for some items to be included in informed consent documents.

subitem not at all important
1
2
3
4
5

essential

Clear selection

Does your paper address subitem X26-ii?
Copy and paste relevant sections from the manuscript (include quotes in quotation marks "like this" to indicate direct quotes from your manuscript), or elaborate on this item by providing additional information not in the ms, or briefly explain why the item is not applicable/relevant for your study

Your answer

“After the names of eligible parents and preteens were passed to the research team, research staff invited parents to participate in a telephone conversation about informed consent with their preteen via email and telephone contacts. The study protocol required up to 3 attempts to reach parents by both email and telephone contact. Staff used an institutional review board (IRB)–approved script to conduct an informed consent procedure with the parent and then with the preteen if the parent provided consent. The script included a description of the intervention and the process of random assignment to a study group, explained the risks and benefits of participation, and reviewed the study activities and incentives for participation. Both parents and preteens were assured that their information was confidential and that participation was voluntary. In addition to preteens who had not received any HPV vaccine, eligible dyads confirmed that they had access to the internet and a mobile device or personal computer to complete the surveys and play the video game. They also provided a mailing address to receive gift certificates. Once they were enrolled, dyads were randomly assigned in a 1:1 ratio to either the intervention group or control group using a simple randomization schedule generated by the study team’s statistician. Those who were assigned to the intervention arm received the game, whereas those assigned to the control group arm did not receive the game. The study procedures were approved by the university’s nonbiomedical.”

X26-iii) Safety and security procedures
Safety and security procedures, incl. privacy considerations, and any steps taken to reduce the likelihood or detection of harm (e.g., education and training, availability of a hotline)

subitem not at all important

1
2
3
4
5X

essential

Clear selection

Does your paper address subitem X26-iii?
Copy and paste relevant sections from the manuscript (include quotes in quotation marks "like this" to indicate direct quotes from your manuscript), or elaborate on this item by providing additional information not in the ms, or briefly explain why the item is not applicable/relevant for your study

Your answer

"For parents and preteens enrolled in Phase 2 and Phase 3 of this study, we will be obtaining informed consent (parents) and assent (preteens) during the screening process. We will only be recruiting and completing interviews with adult parents (n=160) of children 11-12 years old and children 11-12 years old and who are (a) clients(n = 160) in the participating practice group. Research staff will contact the eligible parent/preteen by first email then telephone at a number they supply to the practice (authorization according to HIPAA regulations). Upon answering the phone, the interviewer identifies him/herself, and states the reason for the call. The respondent is then asked about eligibility. An eligible respondent is given the following information: i) an explanation of the purpose of the study, ii) what parents and preteens will do in the study, iii) the duration of the study, iv) that their participation is voluntary; v) that the information collected is confidential, vi) that researcher contact information is available, and vii) that participation in the study or lack of participation will not affect their treatment at their healthcare provider. The participant is then asked if they have any questions prior to giving consent. If the parent agrees, the research staff will then ask to speak with the adolescent directly (currently or a later time when the adolescent is available) to explain the study, read the assent form out loud, allow for questions, and receive his/her verbal assent participate and relevant contact information. If both the parent and child agree to participate, research staff will tell them that an email message with link to the project survey will be to sent for both the parent and preteen. Once they complete the survey, they will be enrolled in the study and assigned to either an intervention or control group."

RESULTS

13a) For each group, the numbers of participants who were randomly assigned, received intended treatment, and were analysed for the primary outcome
NPT: The number of care providers or centers performing the intervention in each group and the number of patients treated by each care provider in each center

Does your paper address CONSORT subitem 13a? *
Copy and paste relevant sections from the manuscript (include quotes in quotation marks "like this" to indicate direct quotes from your manuscript), or elaborate on this item by providing additional information not in the ms, or briefly explain why the item is not applicable/relevant for your study

Your answer

“All practices were asked to screen dyads as part of the intervention. Half of the practices (18/36, 50%) screened at least one dyad, and a total of 131 dyads were referred to the research team. Of these 131 dyads, 16 (12.2%) did not meet the eligibility criteria for the study. Among the 115 that were eligible, 16 (13.9%) refused to participate in the study, and 42 (36.5%) dyads did not respond to repeated email and telephone contact. Almost half of the eligible dyads (55/115, 47.8%) completed the informed consent process and baseline surveys and were enrolled in the study, and 47 dyads completed the postsurvey. The final study sample available for this evaluation of the game consisted of 28 preteens in the intervention group and 27 in the comparison group (21 and 26, respectively, completed the study).”

13b) For each group, losses and exclusions after randomisation, together with reasons

Does your paper address CONSORT subitem 13b? (NOTE: Preferably, this is shown in a CONSORT flow diagram) *
Copy and paste relevant sections from the manuscript (include quotes in quotation marks "like this" to indicate direct quotes from your manuscript), or elaborate on this item by providing additional information not in the ms, or briefly explain why the item is not applicable/relevant for your study

Your answer
The final study sample available for this evaluation of the game consisted of 28 preteens in the

intervention group and 27 in the comparison group (21 and 26, respectively, completed the study).

13b-i) Attrition diagram
Strongly recommended: An attrition diagram (e.g., proportion of participants still logging in or using the intervention/comparator in each group plotted over time, similar to a survival curve) or other figures or tables demonstrating usage/dose/engagement.

subitem not at all important
1
2
3
4
5

essential

Clear selection

Does your paper address subitem 13b-i?
Copy and paste relevant sections from the manuscript or cite the figure number if applicable (include quotes in quotation marks "like this" to indicate direct quotes from your manuscript), or elaborate on this item by providing additional information not in the ms, or briefly explain why the item is not applicable/relevant for your study

Your answer
The final study sample available for this evaluation of the game consisted of 28 preteens in the intervention group and 27 in the comparison group (21 and 26, respectively, completed the study).

14a) Dates defining the periods of recruitment and follow-up

Does your paper address CONSORT subitem 14a? *
Copy and paste relevant sections from the manuscript (include quotes in quotation marks "like this" to indicate direct quotes from your manuscript), or elaborate on this item by providing additional information not in the ms, or briefly explain why the item is not applicable/relevant for your study

Your answer

“A total of 36 practices across 3 regions in North Carolina enrolled in the Protect Them study (Figure 1)—12 practices were recruited from the eastern region during wave 1 (February 2015 to June 2015), 18 practices were recruited from the central region during wave 2 (February 2016 to July 2016), and 6 practices were recruited from the western region during wave 3 (February 2017 to October 2017). All practices were asked to screen dyads as part of the intervention. Half of the practices (18/36, 50%) screened at least one dyad, and a total of 131 dyads were referred to the research team. Of these 131 dyads, 16 (12.2%) did not meet the eligibility criteria for the study. Among the 115 that were eligible, 16 (13.9%) refused to participate in the study, and 42 (36.5%) dyads did not respond to repeated email and telephone contact. Almost half of the eligible dyads (55/115, 47.8%) completed the informed consent process and baseline surveys and were enrolled in the study, and 47 dyads completed the postsurvey. “

14a-i) Indicate if critical “secular events” fell into the study period
Indicate if critical “secular events” fell into the study period, e.g., significant changes in Internet resources available or “changes in computer hardware or Internet delivery resources”

subitem not at all important
1
2
3
4
5X

essential

Clear selection

Does your paper address subitem 14a-i?
Copy and paste relevant sections from the manuscript (include quotes in quotation marks "like this" to indicate direct quotes from your manuscript), or elaborate on this item by providing additional information not in the ms, or briefly explain why the item is not applicable/relevant for your study

Your answer
Consolidation of healthcare practices made recruiting for the study more difficult.

14b) Why the trial ended or was stopped (early)

Does your paper address CONSORT subitem 14b? *
Copy and paste relevant sections from the manuscript (include quotes in quotation marks "like this" to indicate direct quotes from your manuscript), or elaborate on this item by providing additional information not in the ms, or briefly explain why the item is not applicable/relevant for your study

Your answer
N/A

15) A table showing baseline demographic and clinical characteristics for each group
NPT: When applicable, a description of care providers (case volume, qualification, expertise, etc.) and centers (volume) in each group

Does your paper address CONSORT subitem 15? *
Copy and paste relevant sections from the manuscript (include quotes in quotation marks "like this" to indicate direct quotes from your manuscript), or elaborate on this item by providing additional information not in the ms, or briefly explain why the item is not applicable/relevant for your study

Your answer
“ Preteens in the intervention group were 57% (16/28) female and 71% (20/28) white, and in the comparison group, 52% (14/27) were female and 82% (22/27) were white. The comparison group included 5 Blacks or African Americans, and the intervention group included 4 Blacks or African Americans. Two participants in each group were identified as Hispanic. The intervention group contained 9 participants aged 11 years and 19 participants aged 12 years, and the comparison group contained 14 participants aged 11 years, 11 participants aged 12 years, and 2 participants aged 13 years. There were no statistically significant differences between the groups with respect to gender, age, race, and Hispanic ethnicity”

15-i) Report demographics associated with digital divide issues
In ehealth trials it is particularly important to report demographics associated with digital divide issues, such as age, education, gender, social-economic status, computer/Internet/ehealth literacy of the participants, if known.

subitem not at all important
1
2
3
4
5

essential

Clear selection

Does your paper address subitem 15-i? *
Copy and paste relevant sections from the manuscript (include quotes in quotation marks "like this" to indicate direct quotes from your manuscript), or elaborate on this item by providing additional information not in the ms, or briefly explain why the item is not applicable/relevant for your study

Your answer
Small number of participants

16) For each group, number of participants (denominator) included in each analysis and whether the analysis was by original assigned groups

16-i) Report multiple “denominators” and provide definitions
Report multiple “denominators” and provide definitions: Report N’s (and effect sizes) “across a range of study participation [and use] thresholds” [1], e.g., N exposed, N consented, N used more than x times, N used more than y weeks, N participants “used” the intervention/comparator at specific pre-defined time points of interest (in absolute and relative numbers per group). Always clearly define “use” of the intervention.

subitem not at all important
1
2
3
4
5X

essential

Clear selection

Does your paper address subitem 16-i? *
Copy and paste relevant sections from the manuscript (include quotes in quotation marks "like this" to indicate direct quotes from your manuscript), or elaborate on this item by providing additional information not in the ms, or briefly explain why the item is not applicable/relevant for your study

Your answer

“The final study sample available for this evaluation of the game consisted of 28 preteens in the

intervention group and 27 in the comparison group (21 and 26, respectively, completed the study)."

16-ii) Primary analysis should be intent-to-treat
Primary analysis should be intent-to-treat, secondary analyses could include comparing only "users", with the appropriate caveats that this is no longer a randomized sample (see 18-i).

subitem not at all important
1
2
3
4
5X

essential

Clear selection

Does your paper address subitem 16-ii?
Copy and paste relevant sections from the manuscript (include quotes in quotation marks "like this" to indicate direct quotes from your manuscript), or elaborate on this item by providing additional information not in the ms, or briefly explain why the item is not applicable/relevant for your study

Your answer
This is consistent with our analysis.

17a) For each primary and secondary outcome, results for each group, and the estimated effect size and its precision (such as 95% confidence interval)

Does your paper address CONSORT subitem 17a? *
Copy and paste relevant sections from the manuscript (include quotes in quotation marks "like this" to indicate direct quotes from your manuscript), or elaborate on this item by providing additional information not in the ms, or briefly explain why the item is not applicable/relevant for your study

Your answer

HPV Vaccination Initiation Rate. “The vaccine initiation rate was higher in the intervention group than in the control group, but this difference was not statistically significant (22% vs 15%, respectively; P=.31). Vaccination completion rates were also higher in the intervention group than in the control group (9% vs 2%, respectively; P=.10). Although this is not significant, it is noteworthy that only 1 of the 27 comparison group members completed the HPV vaccine series, whereas 5 of 28 intervention group members completed the HPV vaccine series. It should also be noted that most of those http://games.jmir.org/2020/3/e16883/ JMIR Serious Games 2020 | vol. 8 | iss. 3 | e16883 | p. 8 (page number not for citation purposes) JMIR SERIOUS GAMES Cates et al XSL•FO RenderX who initiated were still on schedule to complete, but the date for the next dose had not yet arrived when data were collected.”

“Knowledge and Vaccination Self-Efficacy and Decisional Balance

The mean knowledge score (5 items, range 1-3) was higher in the intervention group than in the comparison group (2.56, SD 0.34 vs 2.28, SD 0.41 respectively; P=.03).

The mean vaccination self-efficacy score was higher in the comparison group than in the intervention group (1.65 vs 1.45, respectively; P=.05). Only 3 of the 8 individual items in the scale were significantly different and were in the direction of confident—not at all: When it is too expensive? When it is too inconvenient?When my friends will know I got the shot? The overall mean decisional balance score trended toward greater support of vaccination, although differences between the groups were not significant. As seen in Table 1, Pros vaccination scores were higher in the intervention group than in the comparison group (2.44 vs 2.31, respectively), and cons vaccination scores were lower in the intervention group than in the comparison group (1.38 vs 1.48, respectively), but again these were not statistically significant differences between the groups.

PENS: The 18 participants who reported on measures of physical/emotional/narrative presence in the game [31] gave mixed reviews on the game. More than half of the participants gave positive scores on game autonomy and competence, ease, and freedom of playing the game (Table 2). At the same time, more than half of the participants called the game boring and said they were not impacted emotionally and that the game did not hold their attention. Thus, the results of this scale revealed both positive and negative evaluations of the game.

Preteens who did not play the game reported that they had technical difficulties (n=2), and a parent determined it was not for me (n=1). Among the preteens who played the game, 78% (14/18) reported that they played it 3 or more times. The majority of players (14/18, 78%) spent more than 10 min on the game at each session. Although the game was designed primarily for

mobile devices (tablets and cellular phones), the majority of the preteens (11/18, 61%) played the game on a personal computer. Half of the participants (9/18, 50%) saved the game to their device. Fifteen of the preteens played the game with a parent, and 4 preteens played with a sibling.

The participants were asked to complete 3 game activities: (1) play a shield game with blue spikey virus balls, (2) find hidden objects in 4 different rooms, and (3) create a potion (Multimedia Appendix 1). Nearly all the preteens (n=16) found hidden objects and created a potion, and 13 played the shield game. The preteens were able to track their progress on a leaderboard, and 12 of them reported that they earned at least one shield on their leaderboard. The game is completed when shields appear on plants in the garden. Less than half of the preteens (n=8) reported that they saw at least one shield in their garden. All but 2 preteens identified the idea behind the secret garden metaphor.

Postintervention Focus Group We conducted 3 focus groups with 7 preteens following each of the 3 waves in the intervention. The preteens generally enjoyed and understood the game, especially playing the hidden objects game and earning points to plant their gardens. They acknowledged that playing the game helped them to be more aware of HPV. Participants were curious about what would happen to them if they were vaccinated. They described the game as “just a game where you… just plant flowers in the garden and make a shield to protect the plants,” and “...it’s a game to help me understand about the HPV shot and what you do in the game.” Participants liked the “hidden figures game... were fun to try to find.” They said the game was easy to play and that it was fun. The preteens remembered that messages appeared in the game, but they could not remember specific messages. Study participation did not impact preteens’ attitudes about HPV vaccination, and they agreed that playing the game made them more aware of HPV as an infection. In terms of designing the next level of the video game, they suggested more hidden objects with a higher level of difficulty and a bigger garden as well as pulling weeds out of the garden to take care of the plants. They would include more activities beyond the shield game and the hidden objects.”

17a-i) Presentation of process outcomes such as metrics of use and intensity of use
In addition to primary/secondary (clinical) outcomes, the presentation of process outcomes such as metrics of use and intensity of use (dose, exposure) and their operational definitions is critical. This does not only refer to metrics of attrition (13-b) (often a binary variable), but also to more continuous exposure metrics such as “average session length”. These must be accompanied by a technical description how a metric like a “session” is defined (e.g., timeout after idle time) [1] (report under item 6a).

subitem not at all important
1
2
3
4

5X

essential

Clear selection

Does your paper address subitem 17a-i?
Copy and paste relevant sections from the manuscript (include quotes in quotation marks "like this" to indicate direct quotes from your manuscript), or elaborate on this item by providing additional information not in the ms, or briefly explain why the item is not applicable/relevant for your study

Your answer
“PENS The 18 participants who reported on measures of physical/emotional/narrative presence in the game [31] gave mixed reviews on the game. More than half of the participants gave positive scores on game autonomy and competence, ease, and freedom of playing the game (Table 2). At the same time, more than half of the participants called the game boring and said they were not impacted emotionally and that the game did not hold their attention. Thus, the results of this scale revealed both positive and negative evaluations of the game.

Of the 21 participants assigned to the intervention group, 86% (18) reported playing the game.

Preteens who did not play the game reported that they had technical difficulties (n=2), and a parent determined it was not for me (n=1). Among the preteens who played the game, 78% (14/18) reported that they played it 3 or more times. The majority of players (14/18, 78%) spent more than 10 min on the game at each session. Although the game was designed primarily for mobile devices (tablets and cellular phones), the majority of the preteens (11/18, 61%) played the game on a personal computer. Half of the participants (9/18, 50%) saved the game to their device. Fifteen of the preteens played the game with a parent, and 4 preteens played with a sibling. The participants were asked to complete 3 game activities: (1) play a shield game with blue spikey virus balls, (2) find hidden objects in 4 different rooms, and (3) create a potion (Multimedia Appendix 1). Nearly all the preteens (n=16) found hidden objects and created a potion, and 13 played the shield game. The preteens were able to track their progress on a leaderboard, and 12 of them reported that they earned at least one shield on their leaderboard. The game is completed when shields appear on plants in the garden. Less than half of the preteens (n=8) reported that they saw at least one shield in their garden. All but 2 preteens identified the idea behind the secret garden metaphor.”

17b) For binary outcomes, presentation of both absolute and relative effect sizes is recommended

Does your paper address CONSORT subitem 17b? *
Copy and paste relevant sections from the manuscript (include quotes in quotation marks "like this" to indicate direct quotes from your manuscript), or elaborate on this item by providing additional information not in the ms, or briefly explain why the item is not applicable/relevant for your study

Your answer
N/A

18) Results of any other analyses performed, including subgroup analyses and adjusted analyses, distinguishing pre-specified from exploratory

Does your paper address CONSORT subitem 18? *
Copy and paste relevant sections from the manuscript (include quotes in quotation marks "like this" to indicate direct quotes from your manuscript), or elaborate on this item by providing additional information not in the ms, or briefly explain why the item is not applicable/relevant for your study

Your answer
N/A

18-i) Subgroup analysis of comparing only users
A subgroup analysis of comparing only users is not uncommon in ehealth trials, but if done, it must be stressed that this is a self-selected sample and no longer an unbiased sample from a randomized trial (see 16-iii).

subitem not at all important
1

2
3
4
5

essential

Clear selection

Does your paper address subitem 18-i?
Copy and paste relevant sections from the manuscript (include quotes in quotation marks "like this" to indicate direct quotes from your manuscript), or elaborate on this item by providing additional information not in the ms, or briefly explain why the item is not applicable/relevant for your study

Your answer
N/A

19) All important harms or unintended effects in each group
(for specific guidance see CONSORT for harms)

Does your paper address CONSORT subitem 19? *
Copy and paste relevant sections from the manuscript (include quotes in quotation marks "like this" to indicate direct quotes from your manuscript), or elaborate on this item by providing additional information not in the ms, or briefly explain why the item is not applicable/relevant for your study

Your answer
N/A

19-i) Include privacy breaches, technical problems

Include privacy breaches, technical problems. This does not only include physical “harm” to participants, but also incidents such as perceived or real privacy breaches [1], technical problems, and other unexpected/unintended incidents. “Unintended effects” also includes unintended positive effects [2].

subitem not at all important
1
2
3
4
5

essential

Clear selection

Does your paper address subitem 19-i?
Copy and paste relevant sections from the manuscript (include quotes in quotation marks "like this" to indicate direct quotes from your manuscript), or elaborate on this item by providing additional information not in the ms, or briefly explain why the item is not applicable/relevant for your study

Your answer
N/A

19-ii) Include qualitative feedback from participants or observations from staff/researchers
Include qualitative feedback from participants or observations from staff/researchers, if available, on strengths and shortcomings of the application, especially if they point to unintended/unexpected effects or uses. This includes (if available) reasons for why people did or did not use the application as intended by the developers.

subitem not at all important
1
2
3
4
5

essential

Clear selection

Does your paper address subitem 19-ii?
Copy and paste relevant sections from the manuscript (include quotes in quotation marks "like this" to indicate direct quotes from your manuscript), or elaborate on this item by providing additional information not in the ms, or briefly explain why the item is not applicable/relevant for your study

Your answer
“Postintervention Focus Group We conducted 3 focus groups with 7 preteens following each of the 3 waves in the intervention. The preteens generally enjoyed and understood the game, especially playing the hidden objects game and earning points to plant their gardens. They acknowledged that playing the game helped them to be more aware of HPV. Participants were curious about what would happen to them if they were vaccinated. They described the game as “just a game where you… just plant flowers in the garden and make a shield to protect the plants,” and “...it’s a game to help me understand about the HPV shot and what you do in the game.” Participants liked the “hidden figures game... were fun to try to find.” They said the game was easy to play and that it was fun. The preteens remembered that messages appeared in the game, but they could not remember specific messages. Study participation did not impact preteens’ attitudes about HPV vaccination, and they agreed that playing the game made them more aware of HPV as an infection. In terms of designing the next level of the video game, they suggested more hidden objects with a higher level of difficulty and a bigger garden as well as pulling weeds out of the garden to take care of the plants. They would include more activities beyond the shield game and the hidden objects.”

DISCUSSION

22) Interpretation consistent with results, balancing benefits and harms, and considering other

relevant evidence
NPT: In addition, take into account the choice of the comparator, lack of or partial blinding, and unequal expertise of care providers or centers in each group

22-i) Restate study questions and summarize the answers suggested by the data, starting with primary outcomes and process outcomes (use)
Restate study questions and summarize the answers suggested by the data, starting with primary outcomes and process outcomes (use).

subitem not at all important
1
2
3
4
5

essential

Clear selection

Does your paper address subitem 22-i? *
Copy and paste relevant sections from the manuscript (include quotes in quotation marks "like this" to indicate direct quotes from your manuscript), or elaborate on this item by providing additional information not in the ms, or briefly explain why the item is not applicable/relevant for your study

Your answer
“**Principal Findings**
HPV Vaccination Initiation Rate The vaccine initiation rate was higher in the intervention group than in the control group, but this difference was not statistically significant (22% vs 15%, respectively; P=.31). Vaccination completion rates were also higher in the intervention group than in the control group (9% vs 2%, respectively; P=.10). Although this is not significant, it is

noteworthy that only 1 of the 27 comparison group members completed the HPV vaccine series, whereas 5 of 28 intervention group members completed the HPV vaccine series. It should also be noted that most of those who initiated were still on schedule to complete, but the date for the next dose had not yet arrived when data were collected.

Of the 36 practices in the study, 18 were able to identify and screen parents and preteens for a total of 55 dyads. The study sample consisted of 21 preteens in the intervention group and 26 preteens in the comparison group who completed the follow-up survey. The objective of our study is to evaluate the acceptability and feasibility of using a serious video game about HPV vaccination with preteens and parents to promote conversations about and decisions to seek HPV vaccination. The scores for preteen HPV vaccination self-efficacy in our study indicated greater support postintervention for the comparison group compared with the intervention group. Only 3 of the 8 individual items were significant and in the direction of lower self-esteem. One plausible explanation for higher scores in the comparison group is that they were less aware of barriers to HPV vaccination, including expense, inconvenience, and their friends knowing that they would get the vaccine. In addition, game play was mostly positive, with more than half of the participants playing the game as intended and wanting to learn more about HPV vaccination. Less positive comments were made about not changing how they felt about the vaccine or not recommending the game to family or a friend. A greater proportion of preteens in the intervention group initiated the vaccine and had higher completion rates than their counterparts in the control group, but these differences did not reach statistical significance.

From our research and that of others, modifiable determinants to increase HPV vaccination for preteens aged 11 to 12 years include (1) knowledge, attitudes, and beliefs of parents, providers, and preteens; (2) parents' concerns that preteen children are too young to receive vaccination and are not sexually active yet, that the vaccine is not safe, and that they do not have a vaccine recommendation from their doctor; (3) preteens' dislike of shots and being minimally involved in the vaccination decision; and (4) providers' concerns about parents' resistance to vaccination, vaccine cost, and duration [33,34]. Gamification has the potential to increase engagement with health messaging relevant to shaping motivation and behavior, such as seeking HPV vaccination [21]. Gamification includes techniques to increase knowledge and shape attitudes about HPV vaccination. These techniques often provoke positive effects, depending on how they are being implemented and used [21]. The use of a garden metaphor, for example, in visualizing the importance of HPV vaccination, facilitates the preteen's conception of a beneficial medical procedure to prevent harmful viruses. In the case of the Land of Secret Gardens, the challenge is to grow a healthy garden, protected from viruses.

The strengths of our study include using a serious video game to motivate interest in HPV vaccination and to promote conversations with parents, family members, and friends. We conducted focus groups with preteens and learned their viewpoints about serious video games. We further conducted focus groups with preteens as we built the game to determine functionality. Finally, we conducted focus groups after game play to learn what worked well and what did not work so well. Our thorough process will help make the game more relevant to the preteens."

22-ii) Highlight unanswered new questions, suggest future research
Highlight unanswered new questions, suggest future research.

subitem not at all important
1
2
3
4
5

essential

Clear selection

Does your paper address subitem 22-ii?
Copy and paste relevant sections from the manuscript (include quotes in quotation marks "like this" to indicate direct quotes from your manuscript), or elaborate on this item by providing additional information not in the ms, or briefly explain why the item is not applicable/relevant for your study

Your answer
Limitations
“The small sample size was a primary weakness of this study. Recruitment and retention were barriers throughout the study. Once clinic staff provided names of potential participants to research staff, follow-up with the parents via our protocol of 3 attempts via both email and telephone contact remained to be a challenge. One obstacle was a high proportion of clients with parents needing informed consent provided in Spanish, an accommodation not available for this study. Another obstacle reflected some of the measurements we had available. For instance, the decisional balance measure was developed with older subjects (college-aged women); therefore, some of the items had to be modified for use in a young population of children aged 11 to 12 years. A further limitation was relying on vaccination and self-efficacy results from a group of older teenagers. This might have skewed the results from younger teenagers.”

“A serious video game on HPV vaccination is acceptable to parents and preteens and can be played as intended. Gamification can be effective in shaping attitudes about the HPV

vaccination. Further research is needed to enhance the game with puzzles and activities that are engaging to the preteen population. "

20) Trial limitations, addressing sources of potential bias, imprecision, and, if relevant, multiplicity of analyses

20-i) Typical limitations in ehealth trials
Typical limitations in ehealth trials: Participants in ehealth trials are rarely blinded. Ehealth trials often look at a multiplicity of outcomes, increasing risk for a Type I error. Discuss biases due to non-use of the intervention/usability issues, biases through informed consent procedures, unexpected events.

subitem not at all important
1
2
3
4
5

essential

Clear selection

Does your paper address subitem 20-i? *
Copy and paste relevant sections from the manuscript (include quotes in quotation marks "like this" to indicate direct quotes from your manuscript), or elaborate on this item by providing additional information not in the ms, or briefly explain why the item is not applicable/relevant for your study

Your answer
This pilot randomized controlled trial is limited to those 11-12 year olds and their parents who choose to enroll in the study offered through their primary care practice providers. The sample

size was small.

21) Generalisability (external validity, applicability) of the trial findings
NPT: External validity of the trial findings according to the intervention, comparators, patients, and care providers or centers involved in the trial

21-i) Generalizability to other populations
Generalizability to other populations: In particular, discuss generalizability to a general Internet population, outside of a RCT setting, and general patient population, including applicability of the study results for other organizations

subitem not at all important
1
2
3
4
5

essential

Clear selection

Does your paper address subitem 21-i?
Copy and paste relevant sections from the manuscript (include quotes in quotation marks "like this" to indicate direct quotes from your manuscript), or elaborate on this item by providing additional information not in the ms, or briefly explain why the item is not applicable/relevant for your study

Your answer
The recruitment of preteens and parents by providers from 36 primary care practices in North Carolina may affect the generalizability of the study, its external validity and applicability to other settings,

21-ii) Discuss if there were elements in the RCT that would be different in a routine application setting
Discuss if there were elements in the RCT that would be different in a routine application setting (e.g., prompts/reminders, more human involvement, training sessions or other co-interventions) and what impact the omission of these elements could have on use, adoption, or outcomes if the intervention is applied outside of a RCT setting.

subitem not at all important
1
2
3
4
5X

essential

Clear selection

Does your paper address subitem 21-ii?
Copy and paste relevant sections from the manuscript (include quotes in quotation marks "like this" to indicate direct quotes from your manuscript), or elaborate on this item by providing additional information not in the ms, or briefly explain why the item is not applicable/relevant for your study

Your answer
The study includes several techniques to promote video game participation (prompts/reminders, more human involvement, training sessions or other co-interventions). Not having these elements could affect participation and attention to gamification use, adoption and outcomes if the intervention is applied outside of a RCT setting.

OTHER INFORMATION

23) Registration number and name of trial registry

Does your paper address CONSORT subitem 23? *
Copy and paste relevant sections from the manuscript (include quotes in quotation marks "like this" to indicate direct quotes from your manuscript), or elaborate on this item by providing additional information not in the ms, or briefly explain why the item is not applicable/relevant for your study

Your answer

ClinicalTrials.gov identifier:NCT04627298

14-1891

24) Where the full trial protocol can be accessed, if available

Does your paper address CONSORT subitem 24? *
Cite a Multimedia Appendix, other reference, or copy and paste relevant sections from the manuscript (include quotes in quotation marks "like this" to indicate direct quotes from your manuscript), or elaborate on this item by providing additional information not in the ms, or briefly explain why the item is not applicable/relevant for your study

Your answer

ClinicalTrials.gov identifier NCT04627298

University of North Carolina, Chapel Hill Protocol Record 14-1891, Normalizing HPV Vaccination in Preteens with a Serious Video Game

25) Sources of funding and other support (such as supply of drugs), role of funders

Does your paper address CONSORT subitem 25? *
Copy and paste relevant sections from the manuscript (include quotes in quotation marks "like this" to indicate direct quotes from your manuscript), or elaborate on this item by providing additional information not in the ms, or briefly explain why the item is not applicable/relevant for your study

Your answer

National Institutes of Health (NIH NIAID 2 R01 AII 13305-05)

X27-i) State the relation of the study team towards the system being evaluated
In addition to the usual declaration of interests (financial or otherwise), also state the relation of the study team towards the system being evaluated, i.e., state if the authors/evaluators are distinct from or identical with the developers/sponsors of the intervention.

subitem not at all important
1
2
3
4
5

essential

Clear selection

Does your paper address subitem X27-i?
Copy and paste relevant sections from the manuscript (include quotes in quotation marks "like this" to indicate direct quotes from your manuscript), or elaborate on this item by providing additional information not in the ms, or briefly explain why the item is not applicable/relevant for

your study

Your answer
The authors/evaluators are distinct from or identical with the developers/sponsors of the intervention. The authors are independent creators and developers of the video game, Land of Secret Gardens.

About the CONSORT EHEALTH checklist

As a result of using this checklist, did you make changes in your manuscript? *

yes, major changes

yes, minor changes

no
No changes.

What were the most important changes you made as a result of using this checklist?

Your answer

How much time did you spend on going through the checklist INCLUDING making changes in

your manuscript *

Your answer
I spent about 2 weeks responding to the CONSORT questionnaire.

As a result of using this checklist, do you think your manuscript has improved? *

yes

no
No,

Other:

Would you like to become involved in the CONSORT EHEALTH group?
This would involve for example becoming involved in participating in a workshop and writing an "Explanation and Elaboration" document

yes

no
No.

Other:

Clear selection

Any other comments or questions on CONSORT EHEALTH

Your answer

STOP - Save this form as PDF before you click submit
To generate a record that you filled in this form, we recommend to generate a PDF of this page (on a Mac, simply select "print" and then select "print as PDF") before you submit it.

When you submit your (revised) paper to JMIR, please upload the PDF as supplementary file.

Don't worry if some text in the textboxes is cut off, as we still have the complete information in our database. Thank you!

Final step: Click submit !
Click submit so we have your answers in our database!

Submit

Never submit passwords through Google Forms.
This content is neither created nor endorsed by Google. Report Abuse - Terms of Service - Privacy Policy
Forms
